# Supplementary figures and images for: Epidemiology of bovine schistosomiasis and associated risk factors in Ethiopia: A systematic review with meta-analysis of published articles, 2008–2018
Source: PLoS One. 2023 Jul 31;18(7):e0283691. doi: 10.1371/journal.pone.0283691 (PMC10389744; doi:10.1371/journal.pone.0283691)

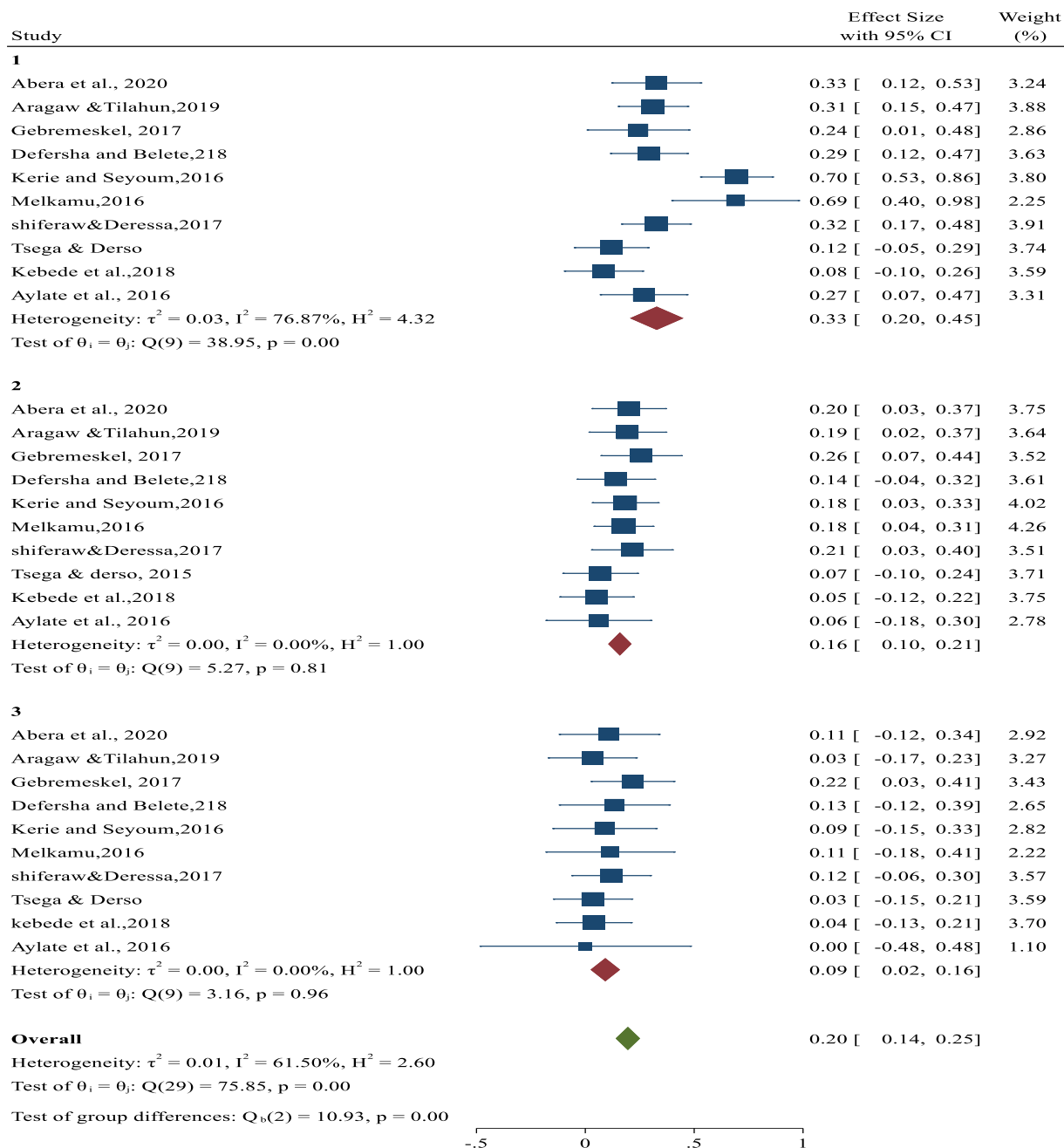

Note: 1 =poor, 2= medium, 3= good

Supplement: S1 Fig — https://drive.google.com/file/d/1xTpipNdNW51PEeLuI0sfTpAcV7wieY9_/view?usp=sharing. (PDF) [file pone.0283691.s001.pdf]

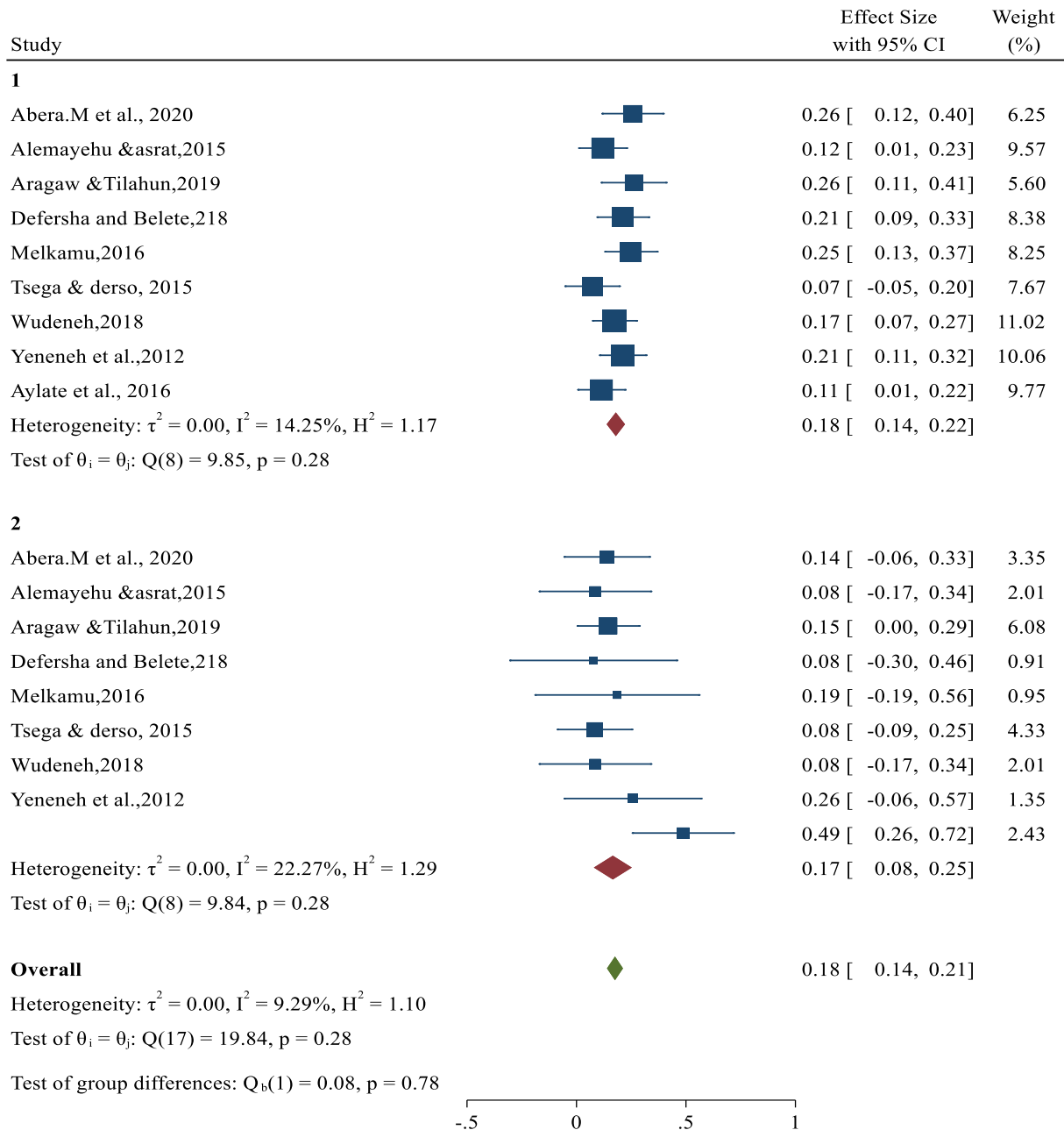

Supplement: S3 Fig — https://drive.google.com/file/d/1ZjTwxBjloIrTbsP7bImgQTZ9t-Q9bzja/view?usp=sharing. (PDF) [file pone.0283691.s003.pdf]

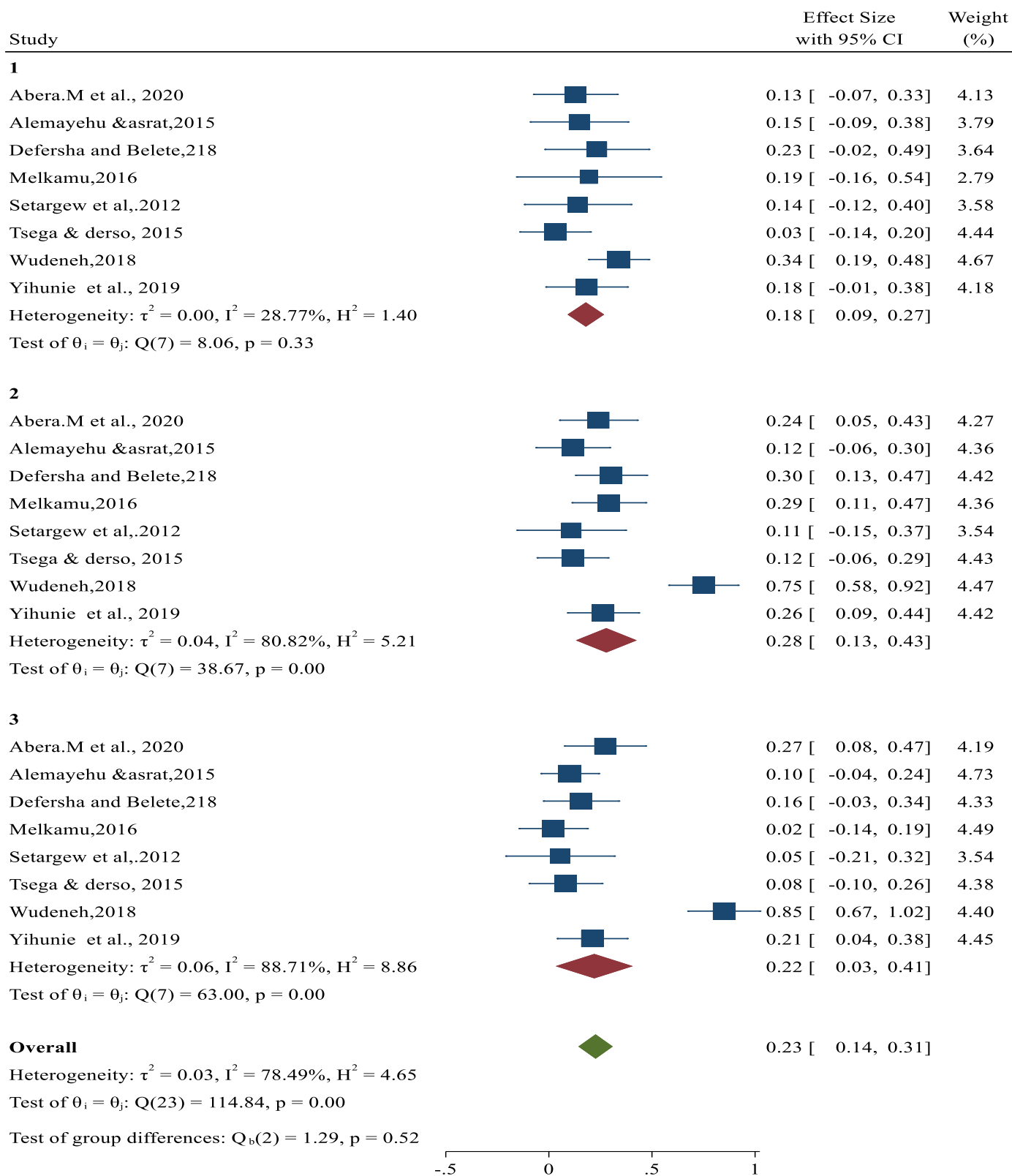

Note: <2 years= 1, 2-5 years= 2, >5 years =3

Supplement: S4 Fig — https://drive.google.com/file/d/1v3yNf9aOhoCMLBHxBDx4bkpoT73vJD17/view?usp=sharing. (PDF) [file pone.0283691.s004.pdf]
