# Supplementary material for: Epidemiology of bovine schistosomiasis and associated risk factors in Ethiopia: A systematic review with meta-analysis of published articles, 2008–2018
Source: PLoS One. 2023 Jul 31;18(7):e0283691. doi: 10.1371/journal.pone.0283691 (PMC10389744; doi:10.1371/journal.pone.0283691)
Supplement: S2 Fig — https://drive.google.com/file/d/1VTlF4kELV_Td49QMVBK-baYycUybW_ek/view?usp=sharing. (PDF) [file pone.0283691.s002.pdf]

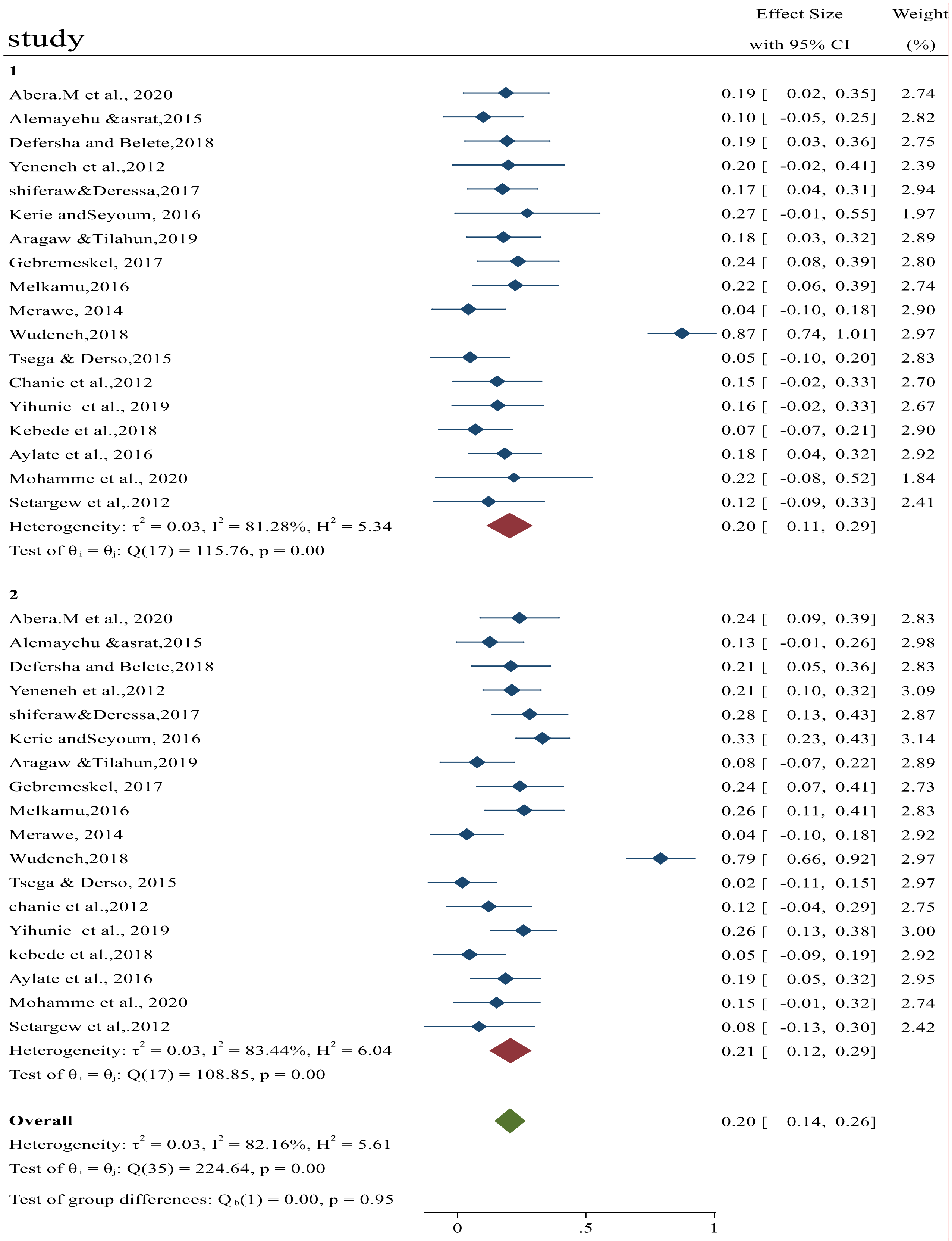

Note: 1= Male . 2= Female
