# Supplementary material for: Epidemiology of bovine schistosomiasis and associated risk factors in Ethiopia: A systematic review with meta-analysis of published articles, 2008–2018
Source: PLoS One. 2023 Jul 31;18(7):e0283691. doi: 10.1371/journal.pone.0283691 (PMC10389744; doi:10.1371/journal.pone.0283691)
Supplement: Attachment — Submitted filename: Reviewers comments in the manuscript.pdf [file pone.0283691.s009.pdf]

PLOS ONE

Epidemiology of bovine schistosomiasis and associated risk factors in Ethiopia: A systematic review with meta-analysis of published articles, 2008-2018

--Manuscript Draft--

**Manuscript Number:**	PONE-D-22-21332
**Article Type:**	Research Article
**Full Title:**	Epidemiology of bovine schistosomiasis and associated risk factors in Ethiopia: A systematic review with meta-analysis of published articles, 2008-2018
**Short Title:**	Epidemiology of bovine schistosomiasis and associated risk factors in Ethiopia
**Corresponding Author:**	Melkie Dagnaw Fenta, Msc<br>UOG: University of Gondar<br>Gondar, Ethiopia ETHIOPIA
**Keywords:**	Bovine schistosomiasis, Ethiopia, Epidemiology, Systematic Review; Meta-Analysis, Risk Factors
**Abstract:**	Schistosomiasis is a tropical and subtropical parasitic infection that affects both animals and people. It's caused by the Schistosoma genus and spreads via snails as an intermediate host. Schistosoma bovis is widely spread in Ethiopia's Northern, Eastern, Southwestern, and Central regions. It is an economically significant cow disease with global health implications. Despite numerous prevalence studies of bovine schistosomiasis in different regions of the country, no systematic review and meta-analysis of the disease have ever been undertaken. As a reason, the purpose of this research was to find out how common bovine schistosomiasis is in Ethiopia, as well as the risk factors connected with it. The eligible studies criteria for Systematic Reviews and Meta-Analyses statement protocol were used to conduct this review. Electronic bibliographic databases such as PubMed, Google Scholar, HINARI, reference list databases, and other manual approaches were employed for literature searches. The pooled prevalence of bovine schistosomiasis and heterogeneity among included studies was estimated by employing random effect models and the inverse variance index, respectively. To investigate heterogeneity across and within studies, subgroup analysis was undertaken based on sample size, study years, and study regions. To assess publication bias and small study effects, funnel plots, Begg's rank test, and Egger's regression test were used. The pooled prevalence was calculated with a 95% confidence interval using STATA 17 software. Based on the inclusion criteria, a total of 20 studies were discovered and included in the meta-analysis. The prevalence of bovine schistosomiasis was found to range from 22 to 45.7%. In this meta-analysis, the estimated pooled prevalence of bovine schistosomiasis was 24% (95% CI: 17% to 31%). The prevalence of bovine schistosomiasis in cattle was shown to be very variable throughout the investigations (I^2 = 88.04%; P = 0.00). In calculating the prevalence of schistosomiasis, the results of the funnel plot, Egger's, and Begg's tests revealed no substantial publication bias (Egger's test; p = 0.509 and Begg's test; P = 0.432). To identify the various risk variables related to the prevalence of bovine schistosomiasis, a pooled odd ratio was used. The pooled odds ratio indicated that poor body condition was positively associated with the infection rate of bovine schistosomiasis (OR: 4.915, 95% CI: 2.675 to 9.030; P =0.00), with poor body conditioned animals having 4.751 times higher likelihood to schistosomiasis infection. This review found that the overall prevalence of bovine schistosomiasis was high and the body condition of animals was identified risk factor for Schistosoma infection among the studied factors. Therefore, the poor body condition of the animals will be warranted. Further, sufficient numbers of prospective studies will be conducted to address other potential risk factors of bovine schistosomiasis in Ethiopia.
**Order of Authors:**	Melkie Dagnaw Fenta, Msc
	Bihonegn Wodajnew Taye, Msc
	Tsegaw Fentie Kassa, Associate professor
	Atsede Solomon Mebratu, Msc
	Firdyawukal Abuhay Tafere, BVSC

Powered by Editorial Manager® and ProduXion Manager® from Aries Systems Corporation

	Nigist Bizu Gessesse, Msc
	Ahmed Abi Abdi Warsame, Msc
**Additional Information:**	
**Question**	**Response**
**Financial Disclosure**<br><br>Enter a financial disclosure statement that describes the sources of funding for the work included in this submission. Review the submission guidelines for detailed requirements. View published research articles from *PLOS ONE* for specific examples.<br><br>This statement is required for submission and **will appear in the published article** if the submission is accepted. Please make sure it is accurate.<br><br>**Unfunded studies**<br>Enter: *The author(s) received no specific funding for this work.*<br><br>**Funded studies**<br>Enter a statement with the following details:<br>• Initials of the authors who received each award<br>• Grant numbers awarded to each author<br>• The full name of each funder<br>• URL of each funder website<br>• Did the sponsors or funders play any role in the study design, data collection and analysis, decision to publish, or preparation of the manuscript?<br>• **NO** - Include this sentence at the end of your statement: *The funders had no role in study design, data collection and analysis, decision to publish, or preparation of the manuscript.*<br>• **YES** - Specify the role(s) played.<br><br>* typeset	no
**Competing Interests**<br><br>Use the instructions below to enter a competing interest statement for this submission. On behalf of all authors, disclose any competing interests that could be perceived to bias this work—acknowledging all financial support and any other relevant financial or non-	The authors have declared that no competing interests exist

financial competing interests.<br><br>This statement is required for submission and **will appear in the published article** if the submission is accepted. Please make sure it is accurate and that any funding sources listed in your Funding Information later in the submission form are also declared in your Financial Disclosure statement.<br><br>View published research articles from *PLOS ONE* for specific examples.<br><br>**NO authors have competing interests**<br><br>Enter: *The authors have declared that no competing interests exist.*<br><br>**Authors with competing interests**<br><br>Enter competing interest details beginning with this statement:<br><br>*I have read the journal's policy and the authors of this manuscript have the following competing interests: [insert competing interests here]*<br><br>* typeset	
**Ethics Statement**<br><br>Enter an ethics statement for this submission. This statement is required if the study involved:<br><br>• Human participants<br>• Human specimens or tissue<br>• Vertebrate animals or cephalopods<br>• Vertebrate embryos or tissues<br>• Field research<br><br>Write "N/A" if the submission does not require an ethics statement.<br><br>General guidance is provided below. Consult the submission guidelines for	N/A

detailed instructions. **Make sure that all information entered here is included in the Methods section of the manuscript.**<br><br>**Format for specific study types**<br><br>**Human Subject Research (involving human participants and/or tissue)**<br>• Give the name of the institutional review board or ethics committee that approved the study<br>• Include the approval number and/or a statement indicating approval of this research<br>• Indicate the form of consent obtained (written/oral) or the reason that consent was not obtained (e.g. the data were analyzed anonymously)<br><br>**Animal Research (involving vertebrate animals, embryos or tissues)**<br>• Provide the name of the Institutional Animal Care and Use Committee (IACUC) or other relevant ethics board that reviewed the study protocol, and indicate whether they approved this research or granted a formal waiver of ethical approval<br>• Include an approval number if one was obtained<br>• If the study involved *non-human primates*, add *additional details* about animal welfare and steps taken to ameliorate suffering<br>• If anesthesia, euthanasia, or any kind of animal sacrifice is part of the study, include briefly which substances and/or methods were applied<br><br>**Field Research**<br><br>Include the following details if this study involves the collection of plant, animal, or other materials from a natural setting:<br>• Field permit number<br>• Name of the institution or relevant body that granted permission	
**Data Availability**<br><br>Authors are required to make all data underlying the findings described fully available, without restriction, and from the time of publication. PLOS allows rare	Yes - all data are fully available without restriction

exceptions to address legal and ethical concerns. See the PLOS Data Policy and FAQ for detailed information.<br><br>A Data Availability Statement describing where the data can be found is required at submission. Your answers to this question constitute the Data Availability Statement and **will be published in the article**, if accepted.<br><br>**Important:** Stating 'data available on request from the author' is not sufficient. If your data are only available upon request, select 'No' for the first question and explain your exceptional situation in the text box.<br><br>Do the authors confirm that all data underlying the findings described in their manuscript are fully available without restriction?	
**Describe where the data may be found in full sentences. If you are copying our sample text, replace any instances of XXX with the appropriate details.**<br><br>• If the data are **held or will be held in a public repository**, include URLs, accession numbers or DOIs. If this information will only be available after acceptance, indicate this by ticking the box below. For example: *All XXX files are available from the XXX database (accession number(s) XXX, XXX.*).<br>• If the data are all contained **within the manuscript and/or Supporting Information files**, enter the following: *All relevant data are within the manuscript and its Supporting Information files.*<br>• If neither of these applies but you are able to provide **details of access elsewhere**, with or without limitations, please do so. For example:<br><br>*Data cannot be shared publicly because of [XXX]. Data are available from the XXX Institutional Data Access / Ethics Committee (contact via XXX) for researchers who meet the criteria for*	All relevant data are within the manuscript and its Supporting Information files.

access to confidential data.<br><br>*The data underlying the results presented in the study are available from (include the name of the third party and contact information or URL).*<br>• This text is appropriate if the data are owned by a third party and authors do not have permission to share the data.<br><br>* typeset	
Additional data availability information:	Tick here if the URLs/accession numbers/DOIs will be available only after acceptance of the manuscript for publication so that we can ensure their inclusion before publication.

Powered by Editorial Manager® and ProduXion Manager® from Aries Systems Corporation

manuscrpit

Click here to access/download;Manuscript;epidemology. edited pm docx.docx

Epidemiology of bovine schistosomiasis and associated risk factors in Ethiopia: A systematic review with meta-analysis of published articles, 2008-2018

Melkie Dagnaw Fenta[1*], Bihonegn Wodajnew Taye[2], Tsegaw Fentie Kassa[3], Atsede Solomon Mebratu[4], Firdyawukal Abuhay Tafere[5], Nigist Bizu Gessesse[6], Ahmed Abi Abdi Warsame[7]

[1, 3,4,5,6] University of Gondar College of Veterinary Medicine, Gondar, Ethiopia. [2]College of Agriculture and Natural Resource, Assosa University, Assosa, Ethiopia and [7]Department of Agriculture and environment science

*melkiedagnaw3528@gmail.com

Abstract

Schistosomiasis is a tropical and subtropical parasitic infection that affects both animals and humans. It's caused by the Schistosoma genus and spreads via snails as an intermediate host. Schistosoma bovis is widely spread in Ethiopia's Northern, Eastern, Southwestern, and Central regions. It is an economically significant cow disease with global health implications. Despite numerous prevalence studies of *bovine schistosomiasis* in different regions of the country, no systematic review and meta-analysis of the disease have ever been undertaken. As a reason, the purpose of this research was to find out how common bovine schistosomiasis is in Ethiopia, as well as the risk factors connected with it. The eligible studies criteria for Systematic Reviews and Meta-Analyses statement protocol were used to conduct this review. Electronic bibliographic databases such as PubMed, Google Scholar, HINARI, and Web of science, reference list databases, and other manual approaches were employed for literature searches. The pooled prevalence of bovine schistosomiasis and heterogeneity among included studies was estimated by employing random effect models and the inverse variance index, respectively. To investigate heterogeneity across and within studies, subgroup analysis was undertaken based on sample size, study years, and study regions. To assess publication bias and small study effects, funnel plots, Begg's rank test, and Egger's regression test were used. The pooled prevalence was calculated with a 95% confidence interval using STATA 17 software. Based on the inclusion criteria, a total of 20 studies were discovered and included in the meta-analysis. The prevalence of bovine schistosomiasis was found to range from 22 to 45.7%. In this meta-analysis, the estimated pooled prevalence of bovine schistosomiasis was 24% (95% CI: 17% to 31%). The prevalence of bovine schistosomiasis in cattle was shown to be very variable throughout the investigations (I^2 = 88.04%; P = 0.00). In calculating the prevalence of schistosomiasis, the results of the funnel plot, Egger's, and Begg's tests revealed no substantial publication bias (Egger's test; p = 0.509 and Begg's test; P = 0.432). To identify the various risk variables related to the prevalence of bovine schistosomiasis, a pooled odd ratio was used. The pooled odds ratio indicated that poor body condition was positively associated with the infection rate of bovine schistosomiasis (OR: 4.915, 95% CI: 2.675 to 9.030; *P*=0.00), with poor body conditioned animals having 4.751 times higher likelihood to schistosomiasis infection. This review found that the overall prevalence of bovine schistosomiasis was high and the body condition of animals was identified risk factor for Schistosoma infection among the studied factors. Therefore, the poor body condition of the animals will be warranted. Further, sufficient numbers of prospective studies will be conducted to address other potential risk factors of bovine schistosomiasis in Ethiopia.

Keywords/Phrases: Bovine schistosomiasis, Ethiopia, Epidemiology, Systematic Review; Meta-Analysis, Risk Factors

Introduction

Schistosomiasis is referred to as bilharziasis after a German physician named Theodor Bilharz, who originally documented the cause of urinary schistosomiasis in 1851 [1]. Schistosomiasis affects productivity, reproduction performances, and medically important parasitic disease of humans and animals, which dwell in the circulatory system of definitive hosts [2].

The major *Schistosoma* species that have a significant impact on domestic and wild animals are found throughout the world's tropical and sub-tropical areas. These include the following: *Schistosoma bovis* parasitizes cattle, goats, and sheep in Africa and Southern Europe; *Schistosoma mattheeii* parasitizes sheep, cattle, and other domestic animals in southern Africa; Schistosoma japonicum parasitizes humans, cats, and mammals in Asia; Schistosoma magrebowiei parasitizes antelopes in Central Africa; and *Schistosoma rodhaini* parasitizes wild rodents and carnivore *Schistosoma spindale, Schistosoma indicum, Schistosoma incognitum, Schistosoma nasale,* and *Schistosoma leiperi* are other Schistosoma species of veterinary interest in Asia [3]. *Schistosoma bovis'* primary hosts are ovines, bovines, and caprines, with small wild ruminants serving as secondary hosts. It's found all over Africa, South West Asia, and Europe [4].

Bovine Schistosomiasis is a trematode infection of cattle caused by the genus Schistosoma, which is spread via snails. *Schistosoma* bovis is endemic in Northern, Southwestern, Eastern, and Central parts of Ethiopia. *Schistosoma bovis* develops a syndrome characterized by liver damage, the roughness of hair coat, pale mucous membrane, severe emaciation, and reduced reproductive performance, resulting in a major economic downturn and a public health problem [5]. Schistosomiasis is one of the fifteen neglected tropical diseases (NTD) because it is unusual amongst helminth diseases for two reasons: much of the pathogenesis is due to the eggs (rather than larvae or adults), and the majority of the pathology is caused by the host's immune system (delayed-type hypersensitivity and granulomatous reactions) [6] and it is the second parasitic disease next to malaria in the human case.

Parasitologic, immunologic, and molecular approaches are used to diagnose Schistosoma infection.
Microscopy is used in parasitological techniques such as the miracidium hatching test and the thick smear test.; immunodiagnostic techniques detect species-specific antigens or antibodies; identification of parasite DNA using molecular methods using the nucleic acid sequence of schistosome GSTs [7], and nucleic acid hybridization techniques using complementary DNA probes were retrieved.
The gold standard approach for detecting fecal egg excretion in the field is the sedimentation technique.

Moisture, rainfall, temperature, and water bodies are examples of environmental influences (stagnant ponds, swamps, streams, rivers, irrigation canals, marshes, and dams), and snail intermediary hosts influence animal and human schistosomiasis [8]. In addition, animal immunity/status, sex, breed, season, age, and farming system play a role in the schistosomiasis infection rate in animals [9].

In Ethiopia, various epidemiological studies on schistosomiasis in cattle were undertaken. The prevalence of *Schistosoma bovis* has been studied by several authors in different periods and different parts of Ethiopia, and a prevalence of 12.3-29% were reported by [10-13] in different districts of the Amhara region. Because the presence of biotypes suitable for the development and multiplication of intermediate hosts is closely linked to the presence of stagnant water bodies and marshy free grazing areas, these epidemiological studies are an indication of the disease's regular occurrence, particularly in the area where stagnant water bodies and marshy free grazing areas.

Schistosomiasis is a poorly understood tropical zoonotic trematode infection that has received little attention. It is a usual process for summarizing results from several studies that are being used largely in human medicine. However, summarizing results is to a more limited extent in veterinary medicine. Besides these limitations, a systematic review of all available studies and a pooled estimate result of various individual studies (meta-analysis) have not yet been conducted in Ethiopia. As a result, the goal of this systematic review and meta-analysis was to offer a pooled prevalence of bovine schistosomiasis and related risk factors based on available cross-sectional research in Ethiopia.

Methods

The PRISMA (Preferred Reporting Items for Systematic Reviews and Meta-analysis) checklist [14] was used to conduct this review. The checklist is used to confirm the inclusion of relevant information from the selected articles based on the underline protocols. The main goal was to figure out the overall prevalence of bovine schistosomiasis in Ethiopia, as well as the associated risk factors.

Search strategy

The literature search and other manuscript preparation were conducted between September 01, 2021, and April 28, 2022. To explore all relevant studies, a thorough search technique was devised. Databases such as PubMed, Google Scholar, HINARI, Web of science, snowball searching from retrieved articles, and other manual methods were used for literature searches to select included studies. This systematic review and meta-analysis used the CoCoPop (Condition, Context, and Population) framework to search for relevant articles. The condition was schistosomiasis (Co), the context was Ethiopia (Co), and the Population was cattle (Pop). The PubMed search strategy included Medical Subject Heading (MeSH) terms and a range of important keywords. Epidemiologically schistosomiasis is a worldwide infection, which infects animals and humans. The research question was "what is the prevalence of schistosomiasis and its associated risk factors among cattle in Ethiopia?"

During an online search, the Boolean operators "AND / OR" were employed to find relevant findings by combining similar phrases /words. Searching terms used were (schistosomiasis OR schistosomiasis infection OR bovine schistosomiasis) AND (epidemiology OR prevalence OR infection rate) AND (cattle OR bovine OR animals infected) and risk factors OR predisposing factors AND (Ethiopia). A restriction was placed on the language of publication is English. All identified studies were imported to EndNote 20 software to remove duplicates and scientific citations of the references.

Study eligibility

The study was conducted according to PRISMA guidelines. The guideline was organized in different sections; firstly arranged systematically based on the title, main objective, and content of the abstract. The second stage involved checking the type of manuscript against a list of inclusion and exclusion criteria. The crucial points to include relevant studies were; (i) all studies have been cross-sectional study design and employed both random and non-random sampling technique, (ii) clear estimation of

prevalence, and (iii) the selection included only prevalence of schistosomiasis on bovine species, (iv) Study animals and the setting described in detail (v) year of study from 2008-2018 conducted in Ethiopia were considered. Articles were selected if they coincide with the above inclusion criteria. Outbreak reports, case series, traditional reviews, cohort studies papers and case-control studies, and experimental (clinical trial) studies were excluded. Finally, these inclusion and exclusion criteria were used for the extraction of data and a meta-analysis (study screening strategy and exclusion reasons) is presented (Fig.1).

Data extraction

The following information was gathered from the included studies, as shown in Table 1. The name of first author and publication year, year of study, study design, study regions and geographical location, sampling design (cross-sectional), sample size, event and non-event, and prevalence schistosomiasis among cattle population.

Study quality assessment

Quality assessment is performed precisely by standard tools because meta-analysis considers the highest level of evidence. Quality assessment was done in this review to check the strength of evidence and methodological quality of the research protocols concerning the review question. Meta-analysis results are dependent on the quality of the included studies. The literature search was done by two researchers independently by considering the quality assessment checklist (PRISMA) [15]. This checklist includes 27 items that include various sections of the article such as the title and abstract, introduction, methods, results, discussion, and other information. The checklist focused on data sources, objectives, study design, sample size, participants, setting, bias, statistical methods, main results, limitations, and funding of the studies and the disagreement was solved by consensus based on the standardized extraction forms to assure consistency and accuracy through discussions by direct contact and telephone conversations.

The following information was extracted and recorded on a Microsoft Excel spreadsheet: first author and ID, title, sample size, participant demographics (sex, age, and body condition), study design (cross-sectional only), study year, and study locations recorded.

Data synthesis and statistical analysis

To determine the weighted-mean estimate of prevalence for *bovine schistosomiasis* across the included studies, a random-effects meta-analysis model with a 95% confidence level was used to pool the studies. STATA version 17 and Comprehensive Meta-Analysis software version 3 were used to conduct the overall meta-analysis (overall effect size, heterogeneity, and weight of each study), subgroup analysis, and pooled prevalence odd ratio (Biostat, Englewood, USA). The Cochran's Q test (reported as the p-value) and inverse variance index (I^2) were used to assess study heterogeneity, which describes the percentage of observed total variation between studies that is due to heterogeneity rather than chance. As explained by Higgins and Thompson [16], the I^2 index was estimated to represent low, moderate, and high heterogeneity, if this corresponds to I^2 values of 25%, 50%, and 75%, respectively. The *p* -values are low suggesting that heterogeneity is present across studies. The presence of heterogeneity between studies was assessed using a forest plot diagram and a Galbraith plot. The forest plot diagram displayed weights, effect sizes, and 95% confidence intervals for individual studies (CLs). Similarly, subgroup analyses for the prevalence of *bovine schistosomiasis* were conducted with 'study year', study region, and sample size to determine the specific variability between studies.

Publication bias is an unavoidable problem in systematic reviews and meta-analyses; it is well known and one of the most important types of bias known as reporting bias, so it is also one of the main threats to meta-analysis validity [17]. Small study effects and publication bias were visualized using funnel plot diagrams, as well as Egger's and Begg's tests [18]. Egger's regression test is used to test the funnel-plot symmetry, and it involves building a regression model with the standardized estimate of the effect size as a dependent variable and the inverse of the standard error (1/SE) as an independent variable. If the intercept deviates significantly from zero, the estimate of the effect is deemed biased [19].

Result

Search results

As viewed in Fig.1, a total of 345 articles were browsed through different electronic databases and with other methods. A total of 186 articles were removed through duplicate, marked as ineligible and other reasons. A total of 79 articles were eliminated through title and abstract screening. Eighty (80) articles reports sought for retrival and 42 evaluated for eligibility. Finally, only a 20 full-text articles for qualitative and quantitative synthesis.

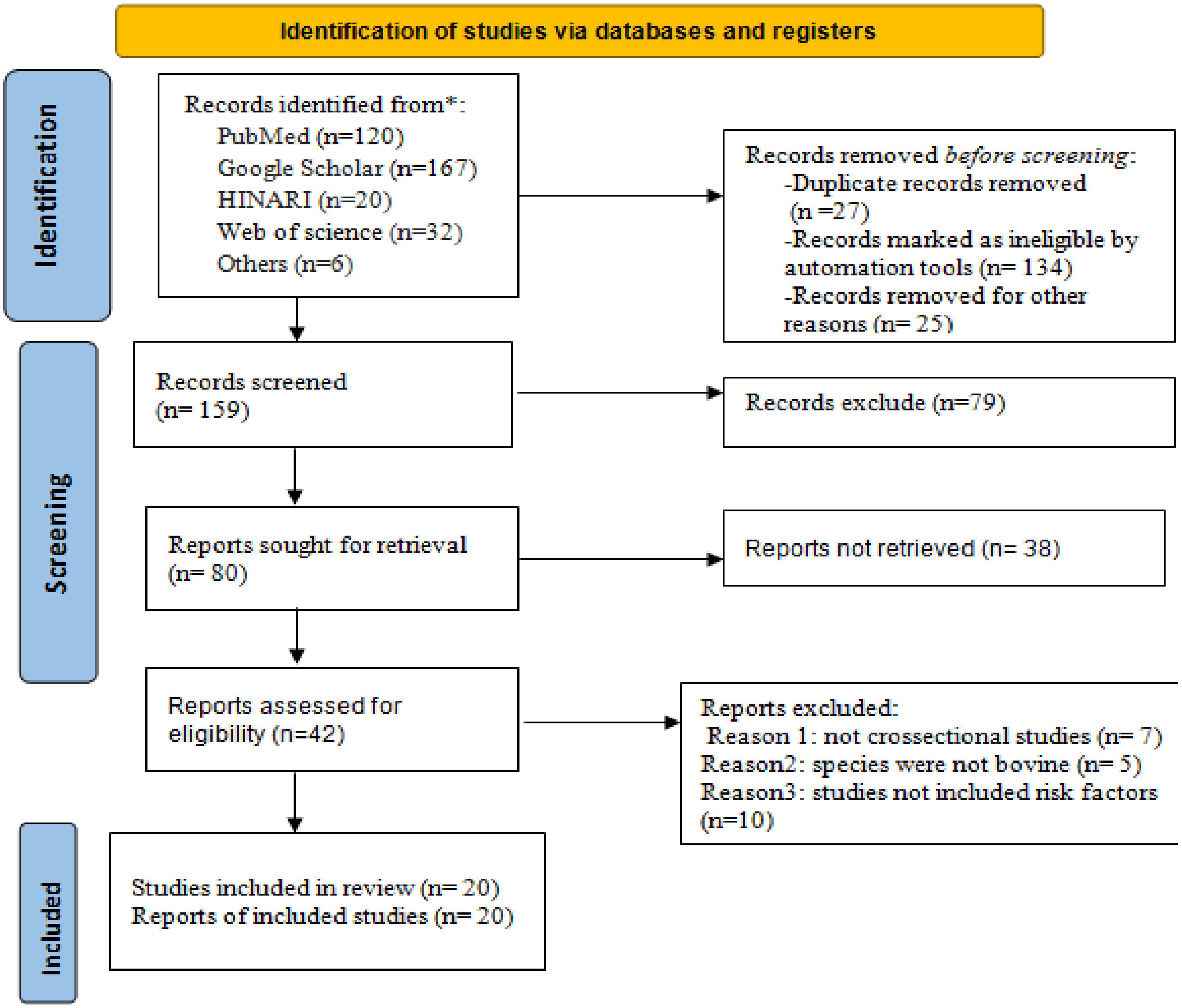

Fig.1 PRISMA flow diagram for selecting included studies in Ethiopia from 2008 to 2018 years.

Characteristics of included studies

Published reports of schistosomiasis infection in cattle in Ethiopia were included in this systematic review. It comprises a total of twenty relevant studies for quantitative as well as qualitative review. A cross-sectional study design was used in all of the included studies. The included studies for this systematic review and meta-analysis were conducted in various parts of Ethiopia between 2008 and 2018 years. Individual studies included in this systematic review had a minimum sample size (cattle) of 167 [20], while the included study had a maximum sample size of 687 [12].

All included studies used coprological examination with sedimentation technique for the recovery of the schistosomiasis eggs (standardized laboratory procedures) indicated by Hansen and Perry, [21]. To evaluate the prevalence rate of bovine schistosomiasis in Ethiopia, 7,378 cattle were used in this case. The apparent prevalence of bovine schistosomiasis ranged between 5.8 and 60.4% [22, 23]. The included studies' detailed characteristics are presented in (Table 1).

Regional distribution of studies

A total of 20 studies were distributed across Ethiopia's five regions (Amhar, Oromia, Afar, Tigray, and SNNPR). The majority of the included studies were from Amhara regional state in northwest Ethiopia, with three studies from Oromia, two from Tigray, and two from Afar Region (Table1). The Amhara region had the highest prevalence (60.4% of bovine schistosomiasis, while the Oromia Region had the lowest (5.8%).

Meta-analysis and bias assessment

The meta-analysis included a total of 20 studies that addressed the prevalence of bovine schistosomiasis. The effect size was calculated using the standardized mean difference and the 95 % confidence interval. On the forest plot, significant heterogeneity was observed across the included studies on bovine schistosomiasis in cattle (I^2 = 88.04 %; P = 0.00), and the pooled effect size was 0.24 (95 % CI: 0.17-0. 31) (Fig.2). Similarly, the heterogeneity and outliers of studies were revealed by Galbraith plot which is the expectation of the studies are within 95% of the area bounded by the two confidence interval lines (Fig. 3**)**.

Table 1 Descriptive summary included studies of the prevalence of bovine schistosomiasis in Ethiopia

Authors	Study Design	Study Year	GL* in Ethiopia	Region	SS	E*	NE*	P*
Abera*et al*., 2020	Cross-sectional	2018	Northwest	Amhara	300	65	235	22
Alemayehu and Asrat, 2015		2014	Northwest	Amhara	384	44	340	11
Aragaw and Tilahun, 2019		2014	Northeast	Afar	369	61	308	17
Chanie*et al*., 2012		2013	Northwest	Amhara	270	37	233	14
Defersha and Belete, 2018		2017	Northwest	Amhara	304	61	243	20
Gebremeskel*et al*, 2017		2011	Northwest	Tigray	389	69	320	18
Habtamu and Wolde, 2016		2010	Northwest	Amhara	687	256	431	37
Kebede*et al*., 2018		2008	SothWest	Oromia	384	22	362	5.8
Kerie and Seyoum, 2016		2016	Northwest	Amhara	532	143	389	26.8
Melkamu, 2016		2012	Northwest	Amhara	400	73	327	18.2
Merawe*et al*., 2014		2014	Southern	SNNPR	252	152	100	60.3
Setargew*et al*., 2012		2010	Northwest	Amhara	167	17	150	10
Shiferaw and Deressa, 2017		2015	Western	Oromia	384	88	296	23
Tsega and Derso, 2015		2013	Northwest	Amhara	384	69	315	18
Wudeneh, 2018		2010	Northwest	Amhara	442	72	370	16.3
Yeneneh*et al*., 2012		2017	Northwest	Amhara	384	232	152	60.4
Yihunie*et al*., 2019		2015	Northwest	Amhara	360	80	280	22.2
Mohammed *et al*., 2020		2015	Southwest	Oromia	179	30	149	16.8
Aylate*et al*., 2017		2016	Northeast	Tigray	405	75	330	18.5
Bertualem*et al*., 2017		2012	Northeast	Afar	400	183	217	45.7

Key: GL* = Geographical Locations, E* = Event, NE* = Non Event, P* = Prevalence in %, Sample size

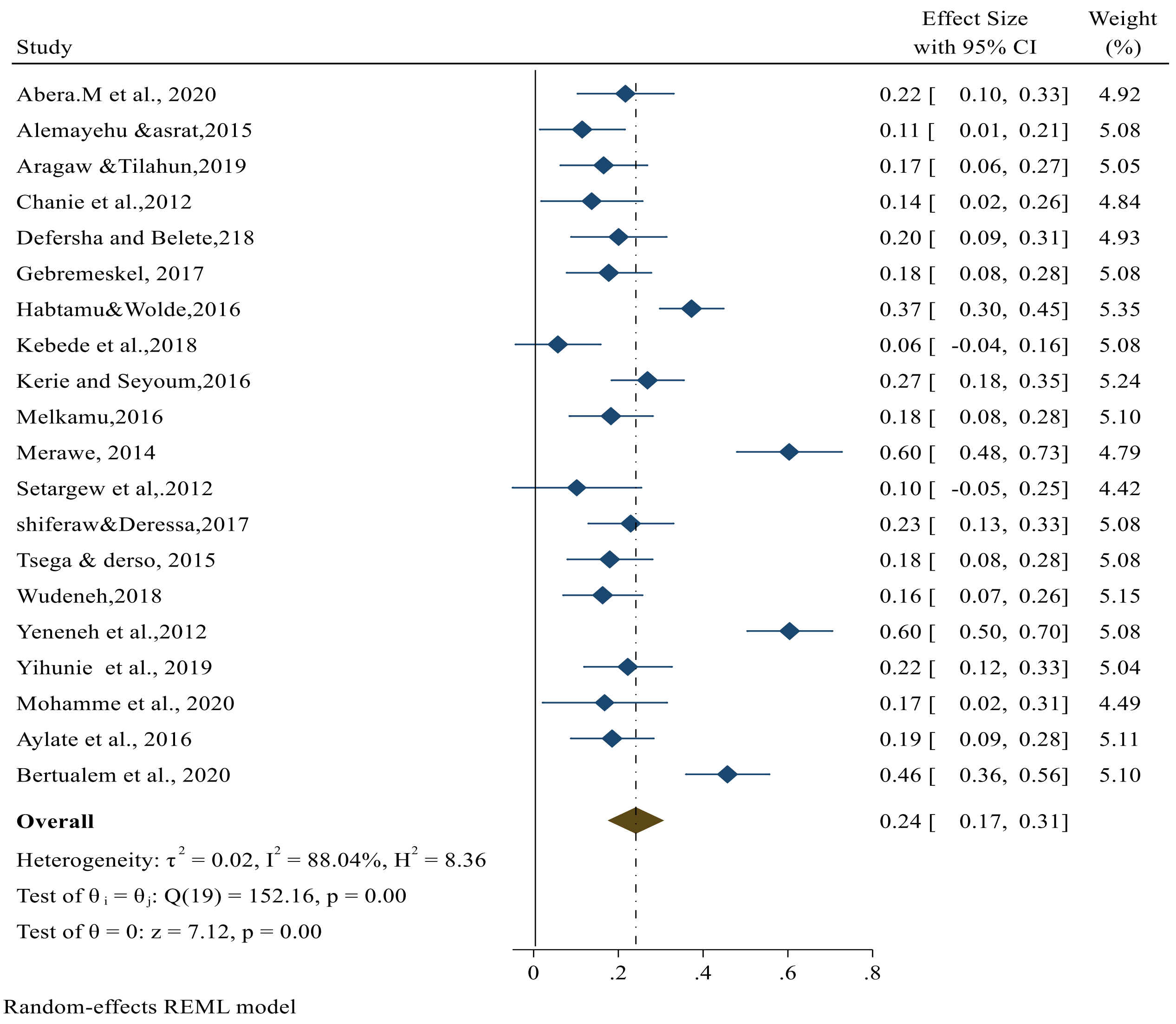

Fig.2 Forest plot for the prevalence of bovine schistosomiasis in Ethiopia.

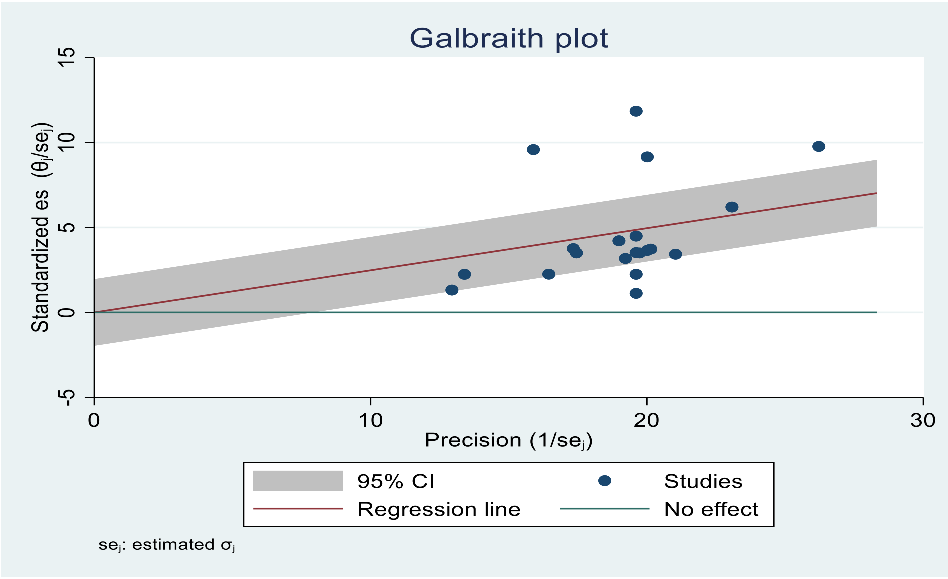

Fi g.3 Galbraith plot portrayed the heterogeneity of included studies for the prevalence of Bovine Schistosomiasis.

As shown above, in Fig. 4, the Galbraith plot confirmed the presence of heterogeneity between studies as almost 95% of studies were not included within the confidence interval. More than eight of 20 studies were not confined to the 95% Confidence interval of the plot.

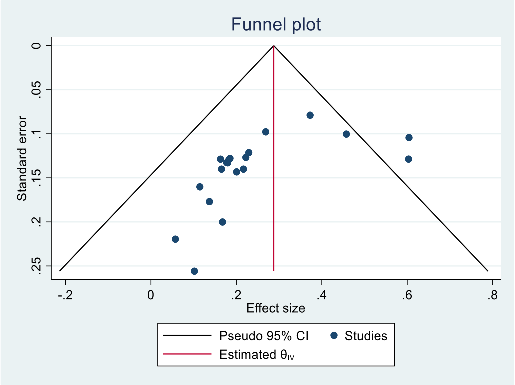

Fig.4 Funnel plot with pseudo 95% confidence limits of pooled prevalence of bovine schistosomiasis in a different region of Ethiopia

The funnel plot (Fig. 4) revealed that there was no asymmetrical distribution of studies and all studies are under a 95% confidence interval which means smaller studies do not tend to be missed.

Subgroup analysis

In light of high heterogeneity, subgroup analysis was conducted using the study year, the sample size, and study site (region) to assess the heterogeneity between studies. Subgroup analysis based on study Region revealed that heterogeneity across studies was greater than sample size or study year. All study years were categorized into three groups (2008-2011), (2012-2014) and (2015-2018), indicating a statistically significant heterogeneity in each group. Similarly, the subgroup analysis by sample size (n=170 - 370), (n=384) and (n=167-687) also revealed substantial heterogeneity between studies. In this subgroup analysis, the highest heterogeneity in the study year and sample size 2012-2014 (I^2=91.78%; *P*=0.00; Fig.7); 170 - 370 (I^2=91.53%; *P*= 0.00; Fig.5) were observed, respectively. The subgroup analysis of bovine schistosomiasis by region also had shown the highest heterogeneity (I^2=93.93%; *P*=0.00; Fig.6) in the Afar region.

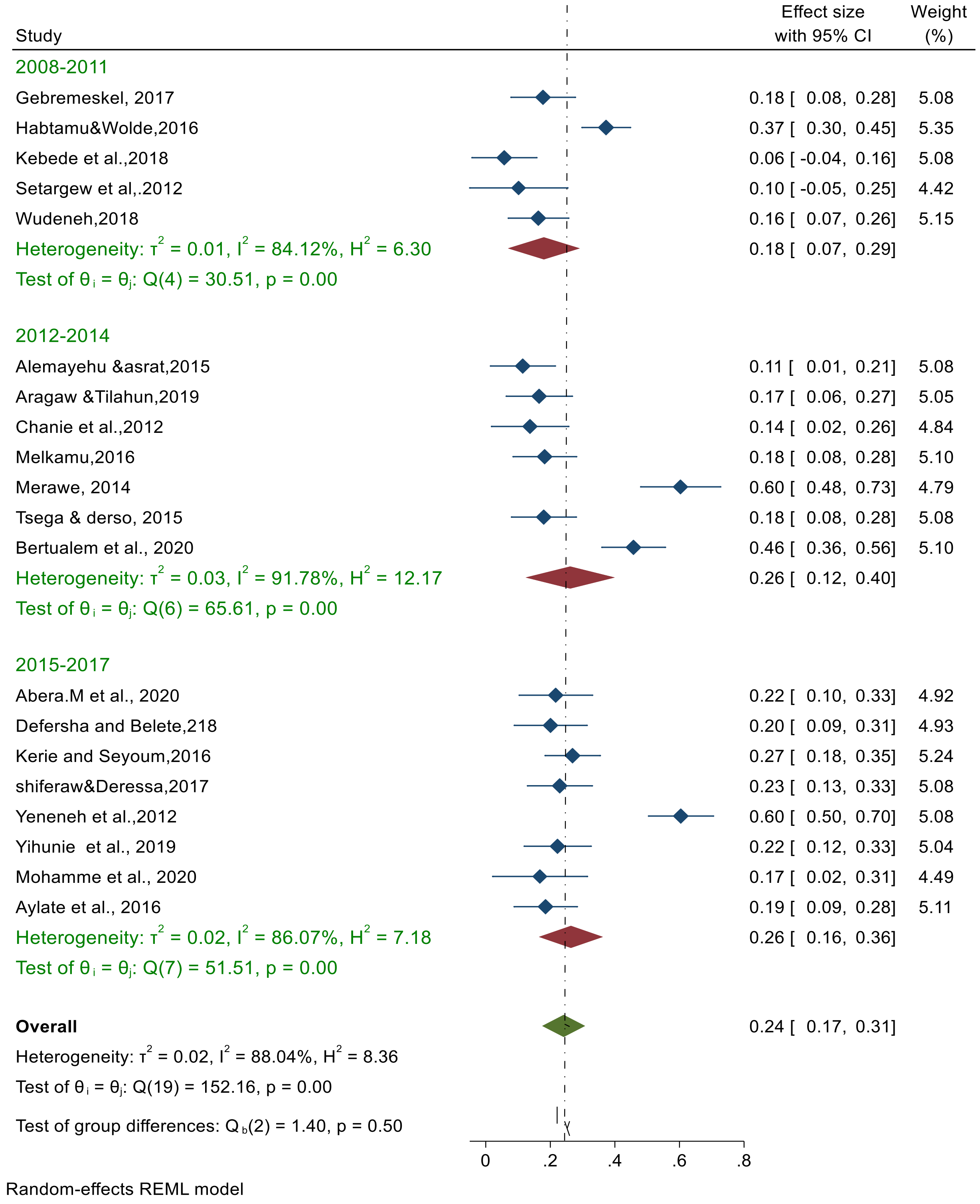

Fig.5 Forest plot for subgroup analysis of the prevalence of bovine schistosomiasis with study year

Table 2 Summary of heterogeneity and Subgroup analysis for comparison of the prevalence of bovine schistosomiasis based on study year, sample size and study regions

**Study year**	**Prevalence (95%CI)**	**Weight (%)**	**I^2**	**tau^2**	**Heterogeneity test *p-value***	**ES***	**Number of studies**
2008-2011	18(0.07, 0.29)	25.07	84.12%	0.01	0.00	0.18	5
2012-2014	26 (0.12, 0.40)	35.04	91.78%	0.03	0.00	0.26	7
2015-2018	26 (0.16, 0.36)	39.89	86.07%	0.02	0.00	0.26	8
**Overall**	24 (0.17, 0.31)	100.00	88.04%	0.02	0.00	0.24	20
**Sample Size**							
N=170-370	28(0.13, 0.42)	38.16	91.52%	0.04	0.00	0.28	8
N=384	15 (0.07, 0.22)	20.32	53.85%	0.02	0.00	0.15	4
N=400-700	26 (0.18, 0.33)	41.17	80.63%	0.01	0.00	0.26	8
**Overall**	24 (0.17, 0.31)	100.00	88.04%	0.02	0.00	0.24	20
**Region**							
Amhara	23(0.15-0.31)	60.2	86.31	0.00	0.00	0.23	12
Oromia	15(0.4-0.26)	14.65	63.26	0.001	0.06	**0.15**	3
Afar	32(0.03-0.6)	10.15	93.9	0.04	0.00	0.32	**2**
Tigray	18(0.11-0.25)	10.19	0.001	0.00	0.91	0.18	**2**
SNNPR	60(0.48-0.73)	4.79	-	-	-	**0.60**	1
**overall**	**0.24(0.17-0.31)**	**100**	**88.04**	**0.02**	**0.00**	0.24	20

ES*= effect size

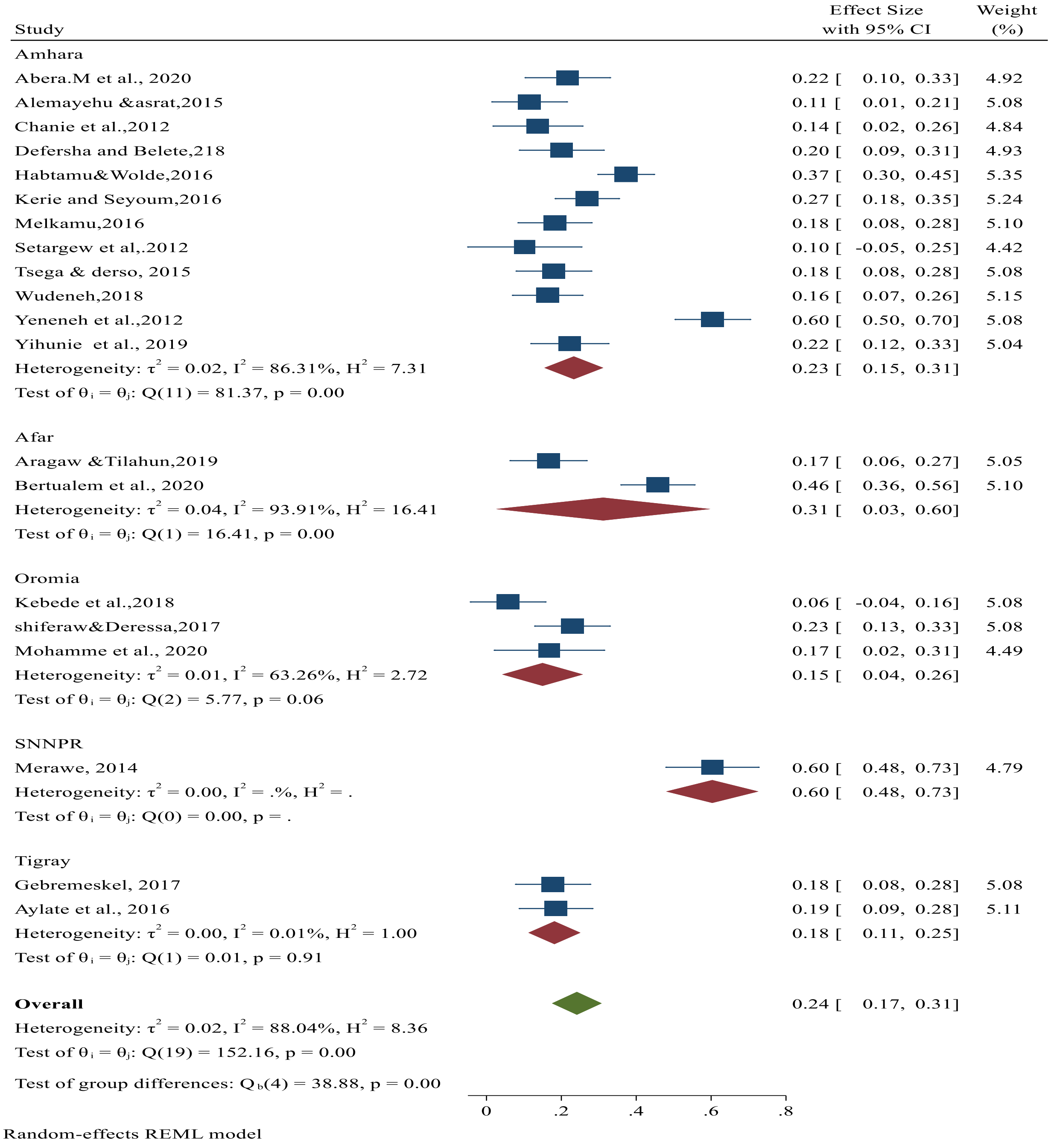

Fig.6 Subgroup analysis of the prevalence of bovine schistosomiasis based on the regional distribution of the studies.

Table 2 shows the subgroup analysis based on sample size, study years, and study regions for comparison of each study to estimate the prevalence of bovine schistosomiasis are significant heterogeneity. Heterogeneity and mean effect size among studies were recorded in the 2012-2014 study year category 0.26 (I^2=91.78%; P = 0.00), whereas the overall heterogeneity was (I^2=88.04%; P=0.00), indicating that there is heterogeneity among the included studies. Similarly, sample sizes were also one factor that causes heterogeneity among the included studies for the prevalence of bovine schistosomiasis. High heterogeneity was investigated in studies having 170-370 sample sizes where (I^2 = 91.52%; P= 0.00).

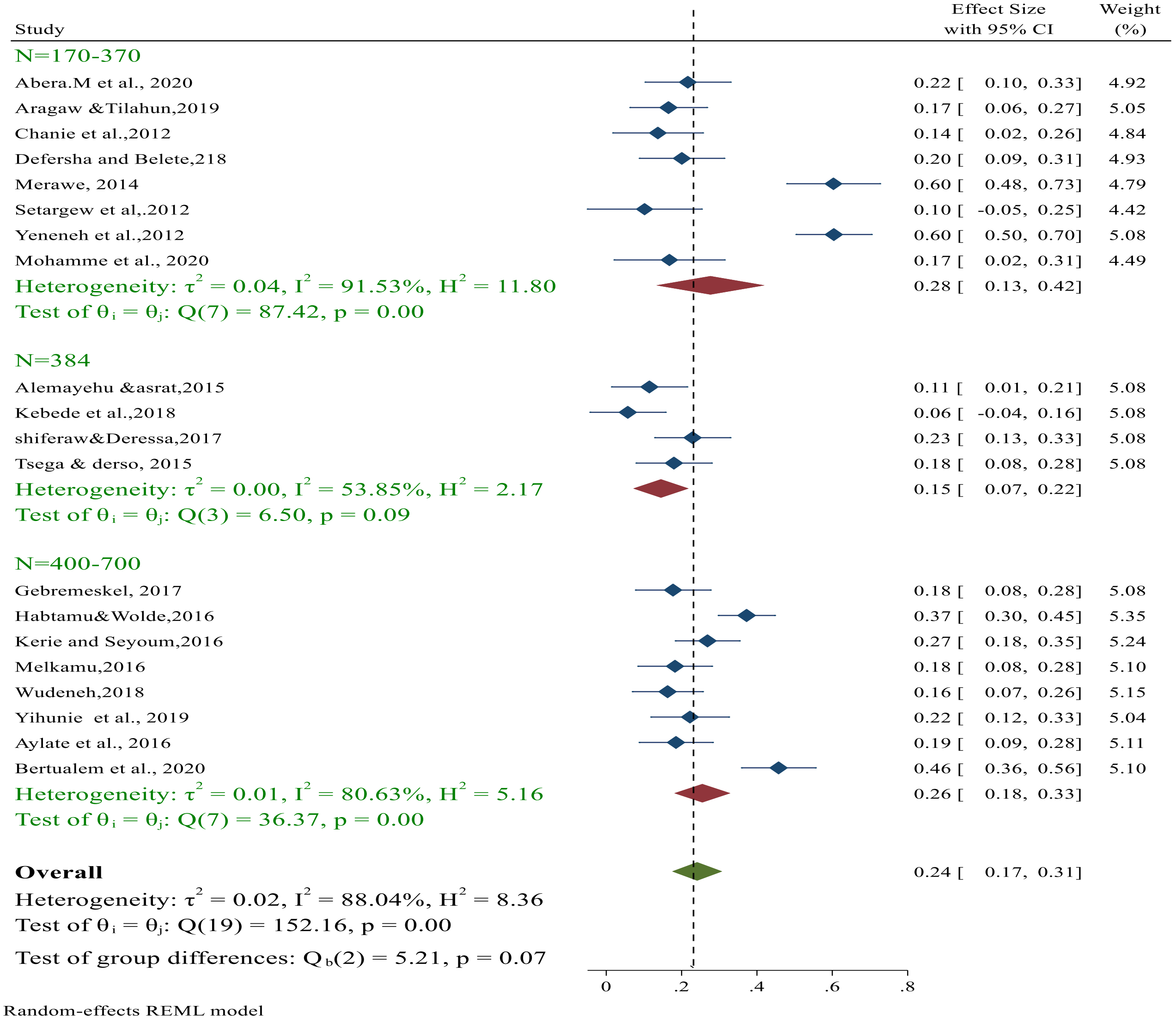

Fig.7 Statistical summary and subgroup analysis based on sample size category

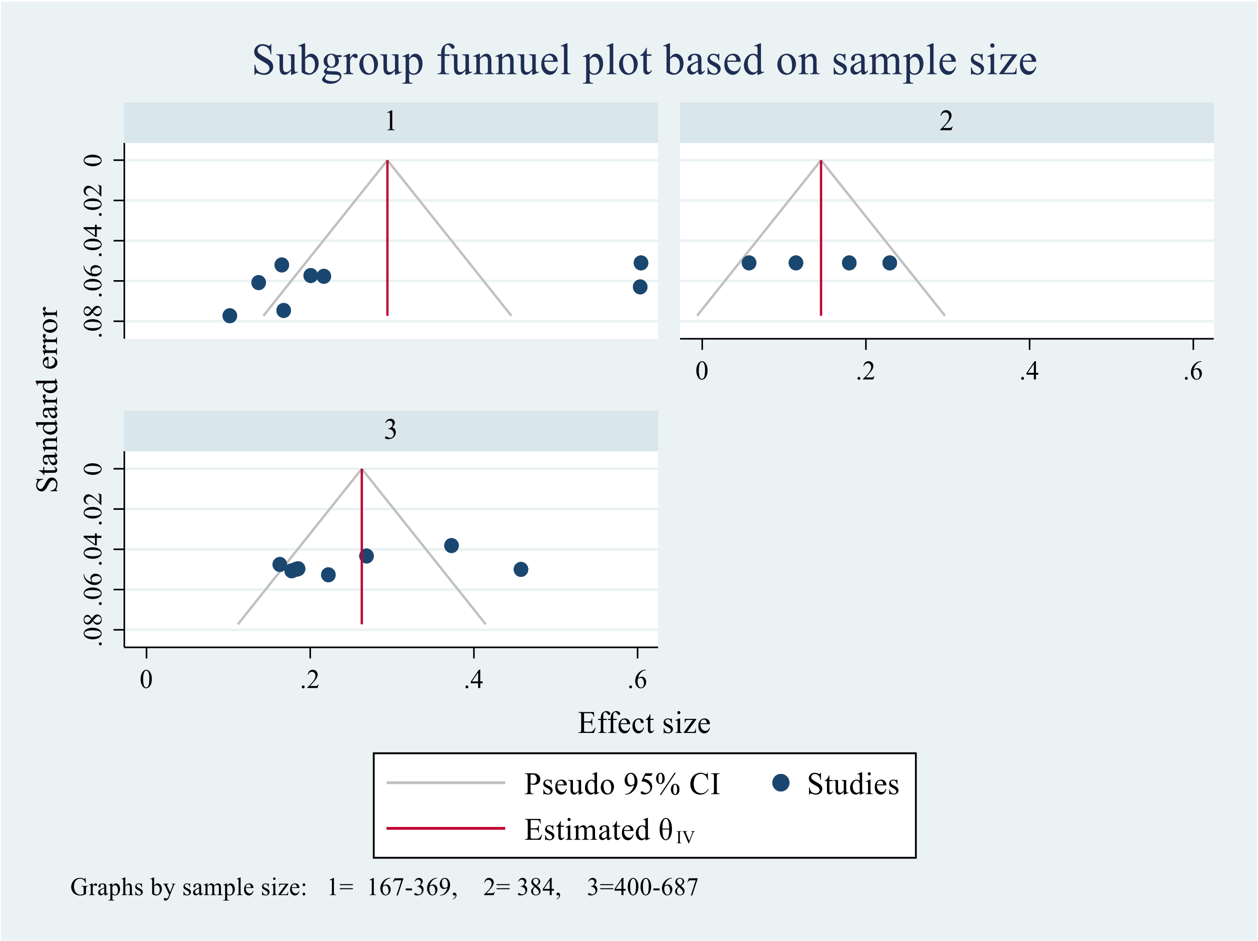

Fig.8 Begg's funnel plot for the subgroup analysis based on sample size for the prevalence of bovine schistosomiasis in Ethiopia

(a)

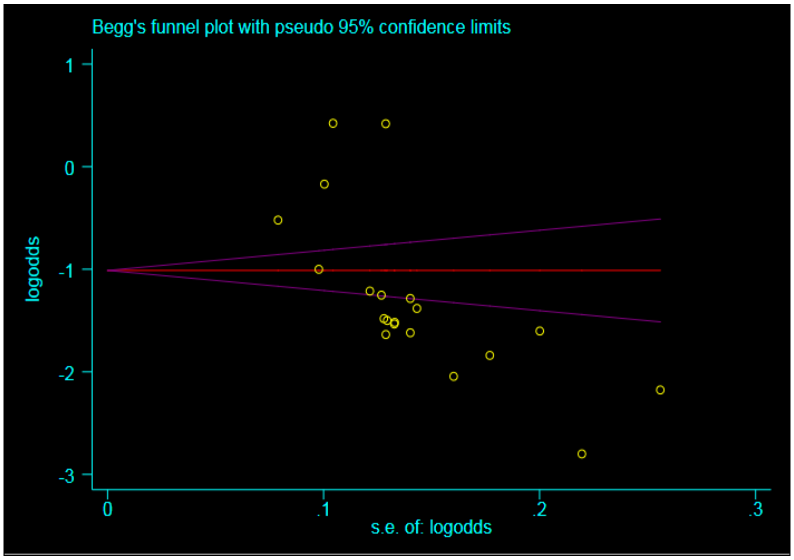

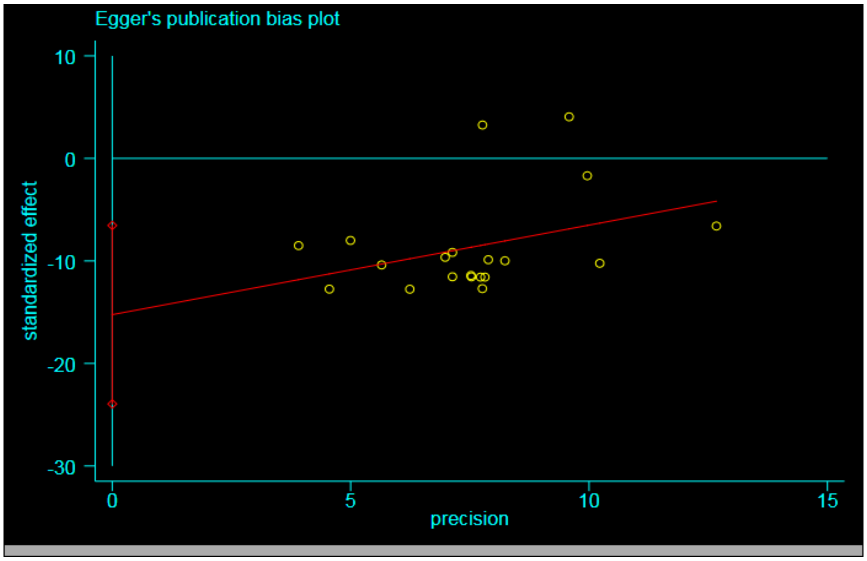

(b)

Fig.9 Begg’s funnel plot (a) and Egger’s plot (b) for publication bias assessment

Figures 8 and 9 shows that there was no publication bias because there was a poor correlation between the effect size and its corresponding variance, while figure 8(b) revealed that there was no publication bias because Egger's test regreses the standardized effect sizes on their precision. As a result, the Egger's test (ET) and Begg's test (BT) plots revealed that there was no statistically significant publication bias in estimating the prevalence of Schistosoma infection in cattle (Egger's test: b = -2.58, 95 percent CI; 0.17-0.31; p = 0.5086 and Begg's test: =0.43196, as shown in (Fig. 9 (a) and (b).

Risk factor studies

Several risk factors have been documented in previous reports in Ethiopia and are presented in (Table 3) & the risk factors considered to affect the occurrence of Schistosoma infection in cattle are geographical locations, age, sex, body condition score (poor, medium or good), breed (cross or local), farm management practices (intensive, semi-intensive or extensive)

Table 3Associated risk factors with bovine schistosomiasis in Ethiopia

Risk factors	Statistically significant studies with risk factors (*p*<0.05)	Not statistically significant risk factors (p>0.05**)**	No of studies
Age	>5 years by Abera*et al*. [24], 2<x < 5 years by Defersha and Belete [25], 2-5 years by Tsega&derso[26], 2-5 years by Wudeneh[27]	Alemayehu and Asrat, [2], Lefevre et al. [11], Setargew*et al*. [20], Melkamu[28], Yihunie*et al*.[29]	8
Sex	Male byAragaw and Tilahun;Wudeneh, [30,27]	Abera *et al*. [24], Alemayehu&asrat [11], Aylate*et al.* [13], Chanie*et al*.[31], Gebremeskel [32], Defersha and Belete [25],Kebede et al.[22],Kerieand Seyoum[10],Habtamu&Wolde[12],Melkamu [28], (Merawe[33], Mohamme*et al*.[34], Tsega&derso[26], shiferaw&Deressa[35], Yihunie*et al*.[29], Setargew*et al*. [20]	18
Body condition	Poor by Abera*et al*. [13,24,25,26, 28,30,35], poor and medium by Kerie and Seyoum[10]	Gebremeskel [32], Kebede*et al*. [22]	10
Breed	cross by Abera*et al*.[24], Local by Aragaw&Tilahun [30] and by Habtamu &Wolde [12]	Alemayehu&asrat[11], Defersha and Belet[25], (Tsega&derso[26], Wudeneh[27],Melkamu[28], Yihunie*et al*.[29]	9
Management system	Extensive by Abera*et a*.[24]Semi-intensive by Aylate*et al*.[13]	Defersha and Belete[25], Melkamu[28]	4

In this systematic review and meta-analysis, we examined the association of different risk factors like body condition, sex, age, and breed and management system with *bovine schistosomiasis*.

Body condition

Out of twenty included studies, ten studies (Table3) addressed the association between body conditions with the occurrence of schistosomiasis among cattle in Ethiopia. Eight studies found a statistically significant relationship between body conditions and the prevalence of bovine schistosomisis, while the remaining two studies found that body conditions did not affect the occurrence of this trematode infection in cattle. So, the highest Prevalence of bovine schistosomisis was studied in poor body conditions [13, 24, 25, 28, 30, 35,] followed by medium body conditions [10] of the animals (Table 2). On the other hand [22, 32] did not find a significant association between the prevalence of schistosomisis and the body condition of the animals which was 24 %(95% CI; 22%-26%; p= 0.79 and 57 %(95%CI 38%-85%; p=0.073), respectively.

Hetrogeneity; A Subgroup meta-analysis of all ten identified studies was performed, the overall mean effect size was 0.20 (95%Cl; 0.14-0.25) and heterogeneity (I^2= 61.50%; p=0.00). The highest variability between studies was seen in poor body conditions of the animals (I^2 =76.87, tau^2=0.03; P= 0.00). In case of medium and good body condition no significance heterogeneity (I^2= 0.00; P=0.81 and I^2= 0.00; p= 0.958) was observed, respectively.

The association of body condition with bovine schistosomiasis

In this meta-analysis, the pooled odds ratio prevalence showed that poor body condition was statistically associated with the prevalence of bovine schistosomiasis (OR: 4.915, 95% CI: 2.675 to 9.030; P=0.00; Fig.10a) showed that animals in poor body condition had a 4.751 times higher risk of bovine schistosomisis than animals in good body condition. Similarly, medium body score was positively associated with the prevalence of bovine schistosomiasis (OR: 1.757, 95 percent CI: 1.301 to 2.373; P=0.00; Fig. 10b), indicating that animals in poor body condition were 1.757 times more likely to be infected than animals in good body condition.

a)

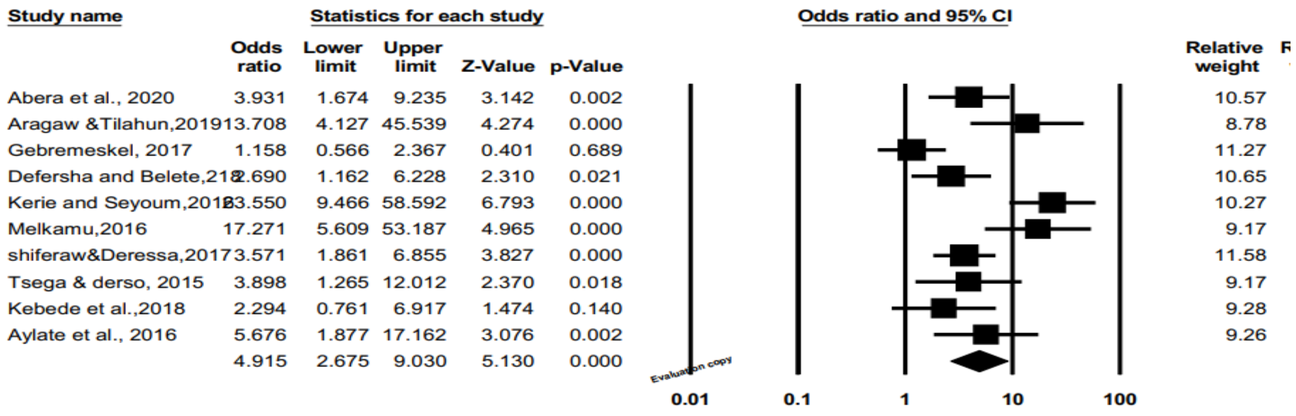

b)

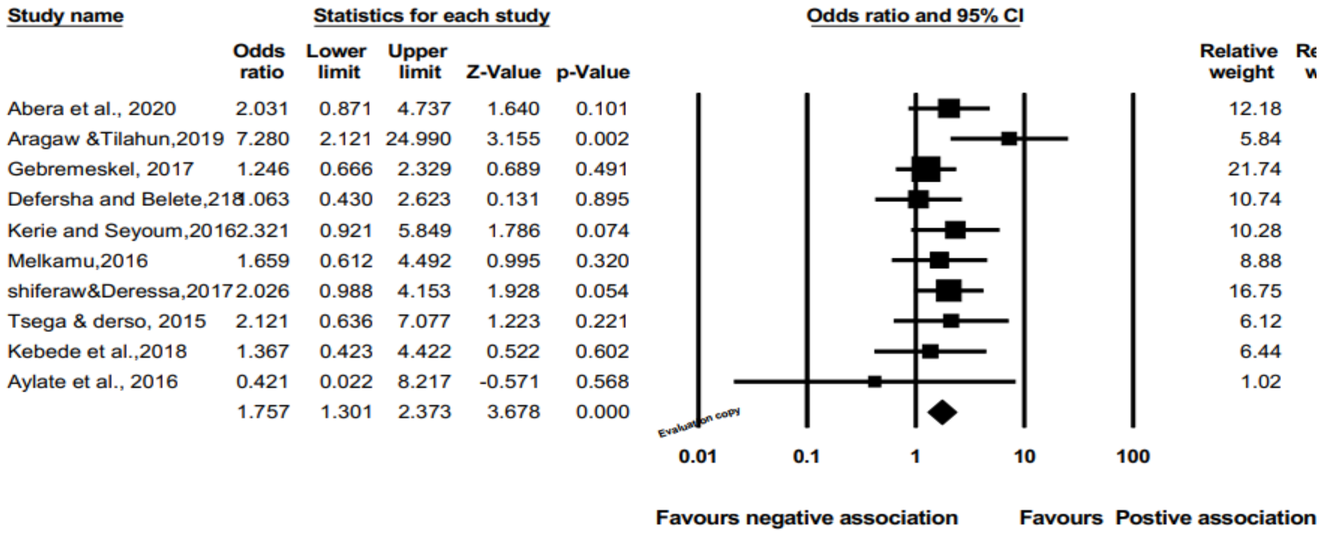

Fig. 10 (a) and (b) Forest plots of odds ratio for the association of poor vs. good body condition (a); medium vs. good body condition (b) with the prevalence of bovine schistosomiasis

Publication bias; detailed examination of the funnel plot and the Egger regression test were used to assess publication bias. The funnel plot revealed that there was no asymmetrical distribution of articles (Fig.11), and most studies are inside the Pseudo 95% confidence interval which depicts that smaller studies do not tend to be missed to be reported to the scientific community. Similarly, the linear extrapolation Egger test for small-study effects revealed no statistically significant publication bias in estimating the prevalence of *schistosomisis* infection related to body condition in cattle (Egger's test: b = 0.71, 95% CI; 0.14 to 0.218; *P*= 0.5246) as indicated in(Fig.11).

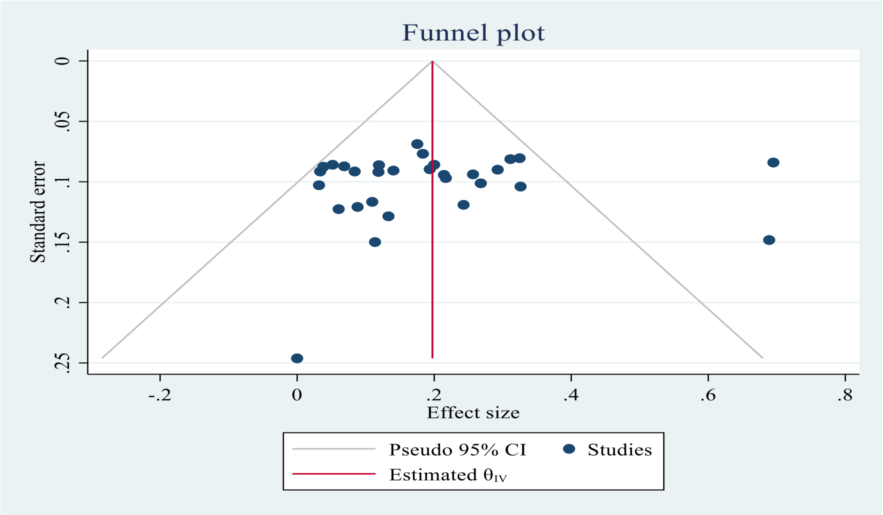

Fig. 11 Funnel plot for publication bias assessment between studies by animal body condition.

The association of sex vs. prevalence of bovine schistosomiasis

A total of eighteen studies addressed the association between the prevalence of bovine schistosomiasis and the sex of the animals (Table 3). Significant findings were reported in only two studies, there was a positive association between the male gender and the prevalence of bovine schistosomisis [27, 30] whereas sixteen studies showed no significant association between the occurrence of bovine schistosomisis with sex.

Meta-analysis of eighteen included studies was conducted to examine the association between prevalence of bovine schistosomiasis with sex of animals, pooled prevalence odd ratio of sexes were performed in bovine schistosomiasis among cattle population. As shown in figure (12), the sex of animals was not significant association (pooled OR=1.007, 95%Cl 0.867 to 1.170; p>0.05) with the prevalence of schistosomiasis

Hetrogeneity assesment: Subset meta-analysis was carried out based on gender (1=male, 2=female).The overall effect size was 0.20 (95% Cl; 0.14 to 0.26) and heterogeneity (I^2= 82.16%; p= 0.00). The higher variability was seen in female animals (I^2= 83.44, p= 0.00) compared with male animals (I^2 =81.28%; p= 0.00 (Table 4). So, the current meta-analysis result showed that heterogeneity was statically significant among the studies of bovine schistosomiasis with the sex of the animals.

Study name	Odds ratio	Lower limit	Upper limit	Z-Value	p-Value	Relative weight
Abera.M et al., 2020	0.732	0.419	1.280	-1.094	0.274	7.24
Alemayehu &asrat,2015	0.768	0.401	1.473	-0.793	0.428	5.33
Defersha and Belete,2018	0.914	0.519	1.607	-0.314	0.754	7.08
Yeneneh et al.,2012	0.919	0.498	1.696	-0.269	0.788	6.01
shiferaw&Deressa,2017	0.542	0.333	0.881	-2.469	0.014	9.55
Kerie andSeyoum, 2016	0.752	0.384	1.476	-0.828	0.408	4.97
Aragaw &Tilahun,2019	2.636	1.359	5.112	2.869	0.004	5.14
Gebremeskel, 2017	0.960	0.559	1.651	-0.146	0.884	7.69
Melkamu,2016	0.828	0.486	1.410	-0.696	0.486	7.96
Merawe, 2014	1.187	0.422	3.341	0.325	0.745	2.11
Wudeneh,2018	1.832	1.097	3.058	2.315	0.021	8.59
Tsega & Derso, 2015	2.769	0.819	9.359	1.639	0.101	1.52
Chanie et al.,2012	3.455	1.409	8.472	2.709	0.007	2.80
Yihunie et al., 2019	0.535	0.303	0.946	-2.152	0.031	6.96
Kebede et al.,2018	1.561	0.651	3.742	0.998	0.318	2.95
Aylate et al., 2016	0.981	0.594	1.620	-0.076	0.940	8.96
Mohamme et al., 2020	1.567	0.654	3.753	1.008	0.313	2.96
Setargew et al,.2012	1.507	0.545	4.169	0.790	0.430	2.18
	1.007	0.867	1.170	0.091	0.927	

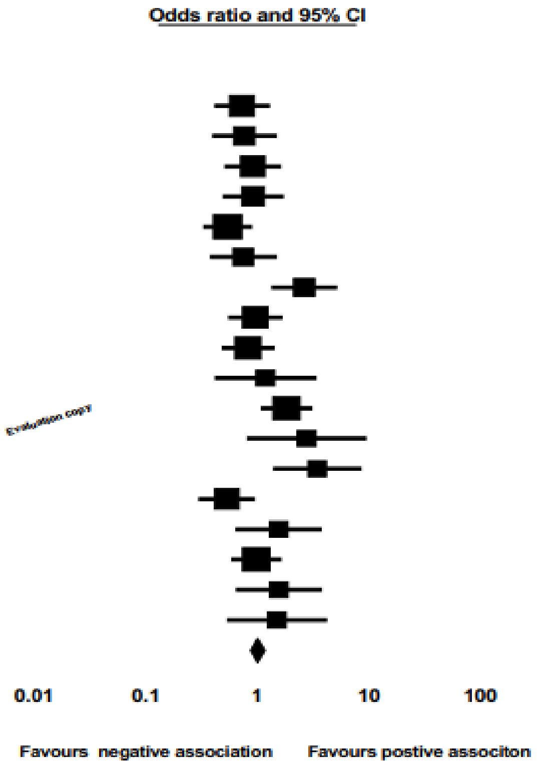

Fig.12 Forest plots of odds ratio for the association of male vs. female with the prevalence of bovine schistosomiasis

Publication bias; detailed examination of the funnel plot and the Egger regression test, which is sex as the moderator by using the random effect model and restricted maximum likelihood methods, were used to assess publication bias (REML). The funnel plot revealed that there was no asymmetrical distribution of articles (Fig. 13), and most studies are inside the Pseudo 95% confidence interval which depicts that smaller studies do not tend to be missed to be addressed by the scientific community. Furthermore, the results of the linear extrapolation Egger test for small-study effects revealed that there was no statistically significant publication bias in estimating the prevalence of *Schistosoma* infection related to the sex of animals (Egger's test: b = -1.50, 95% CI; 0.14 to 0.26; p= 0.579).

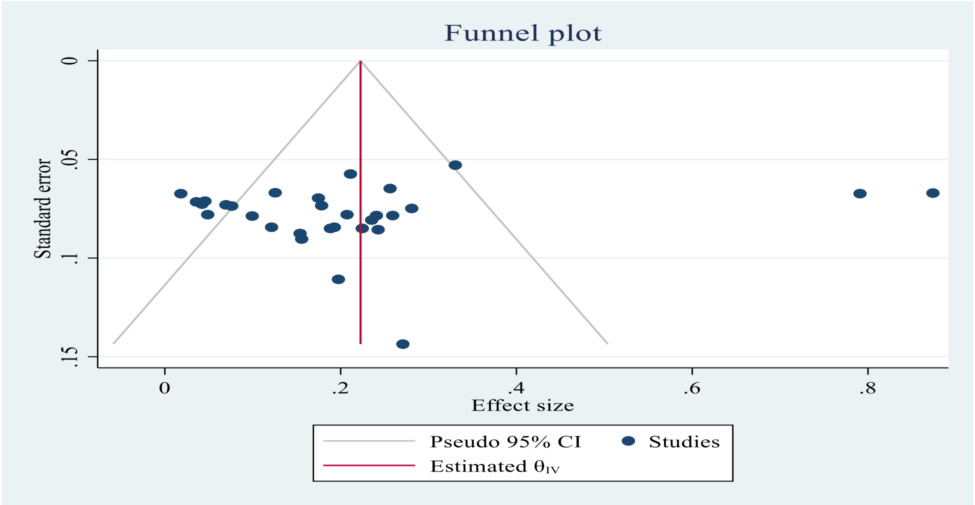

Fig.13 Funnel plot for publication bias assessment articles of the prevalence of bovine schistosomiasis based on sex

Association breed with schistosomiasis

From previous reports, nine studies addressed the association between breed and the occurrence of schistosomisis among cattle in Ethiopia depicted as (Table3). Three studies found a significant association between breed and the prevalence of *bovine schistosomisis* [12, 24, 30]. These studies suggested that bovine schistosomisis was more prevalent in local than the cross breed of cattle. However, six studies suggested no significant association between the prevalence of schistosomisis and the breed of the animals [11, 23, 25-28].

So, to avoid such ambiguous reports, a meta-anlysis of pooled prevalence odd ratio examined the association of age with *bovine schistosomiasis* by using a random effect model. Hence, the pooled meta-analysis revealed no significant difference in the cross and local breed of cattle with the odds ratio of 1.310 (95%: Cl 0.738 to 2.324; *P*= 0.356; Fig.14) with the prevalence of *bovine schistosomiasis*.

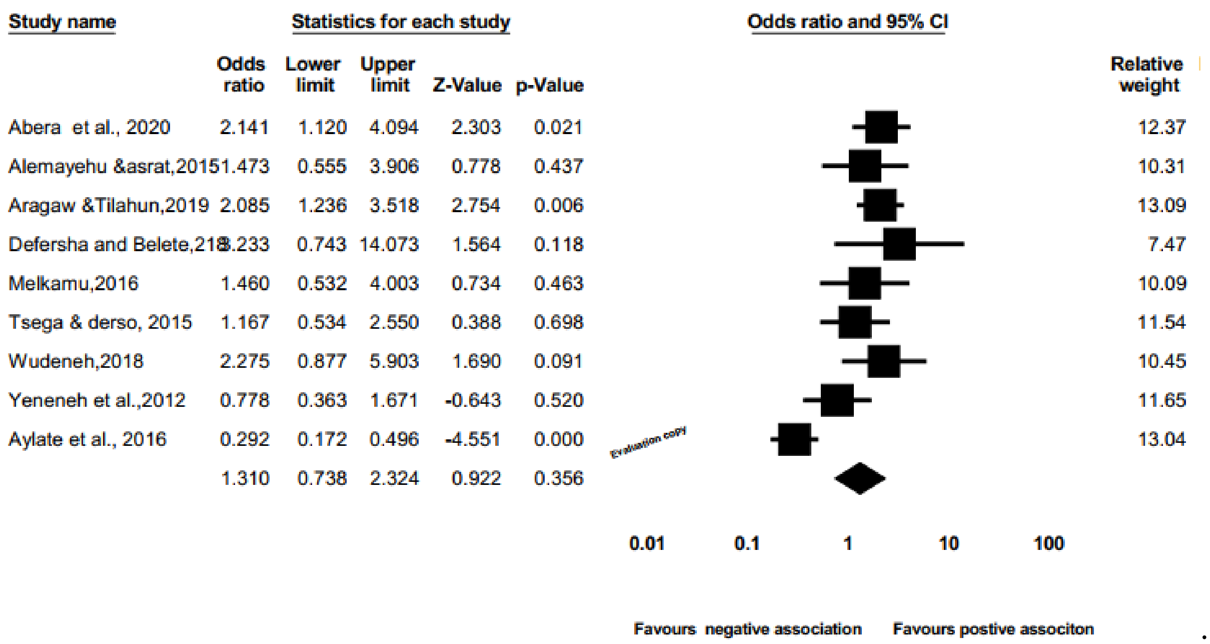

Fig.14 Forest plots of odds ratio for the association of local vs. cross breed with the prevalence of bovine schistosomiasis

Hetrogeneity; based on the nine identified studies, a meta-analysis was conducted to investigate heterogeneity within and across studies using a random effect model. For studies reporting the breed-effect on the prevalence of *bovine schistosomiasis*, there was no strong evidence of between-study heterogeneity (I^2=9.29%, p = 0.28; (Table 4); the pooled effect estimate was 0.18 (95%CL: 0.14 to 0.21) in random effect meta-analysis.

Assessment of bias; detailed examination of the funnel plot was used to assess publication bias, which was confirmed by the Egger regression test. The funnel plot revealed that there was no asymmetrical distribution of articles (Fig.15), and the majority of studies are within the Pseudo 95% confidence interval. The distribution did not suggest that more extreme findings were being selectively published. Likewise, the results of the regression-based Egger test for small-study effects showed no significant (Egger's test: b = 0.18, *p* = 0.7902) publication bias in estimating the prevalence of schistosomisis infection in a breed of cattle.

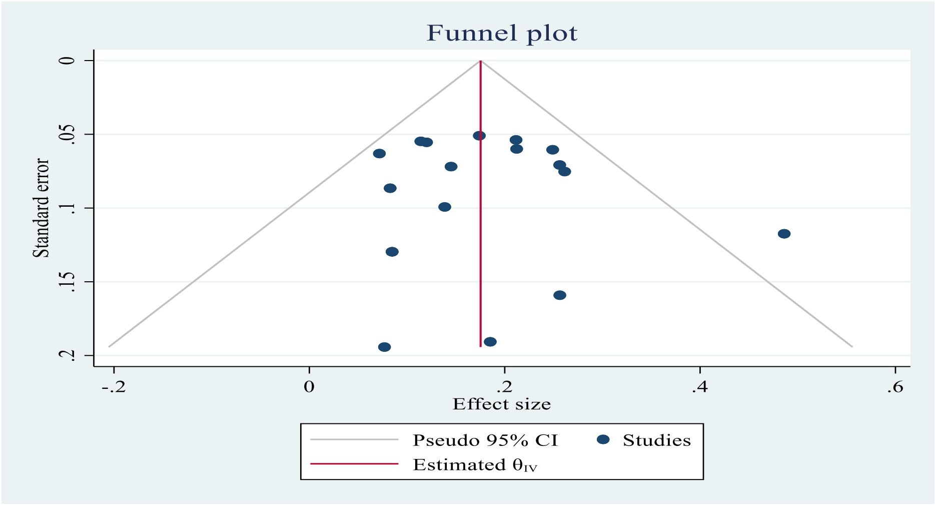

Fig.15 Funnel plot for publication bias assessment of the prevelence of bovine schistosomiasis by breed type

Association of age with schistosomiasis

Eight of the twenty studies examined the relationship between the prevalence of schistosomisis and different ages of the animals in the previous report, discovered a statistically significant association in four of the eight cases by Abera *et* al. [24], (>5 years)& 2- 5 years by Tsega&derso; Defersha and Belete; Wudeneh), [25-27] and other four studies were not reported a significant association between the prevalence of schistosomisis and age of the animal) [11,20,23,28]. According to these findings, the prevalence of schistosomiasis was highest in the 2-5 years age category of animals followed by>5 years of age of animals.

Consequently, meta-analyses of the pooled odds ratio of eight studies were conducted to explore the association between different age categories of animals with bovine schistosomiasis. As shown in figure (16), in three age categories (<2, 2-5, and >5 years) of animals were not shown significant association with the odds ratio of 1.007 (95%; 0.867 to 1.170; Fig.16 (a)), 1.245 (95%; 40.434 to 3.571; Fig.16 (b)) and 1.587 (95%; 0.893 to 2.822; Fig. 17(c)) and p>0.05, respectively. This pooled prevalencestudy is similar from the included studies [11, 20, 23] and contradict with (>5 years [24] & 2- 5 years [25- 27].

a)

Study name	Odds ratio	Lower limit	Upper limit	Z-Value	p-Value	Relative weight
Abera.M et al., 2020	0.467	0.221	0.989	-1.989	0.047	13.24
Alemayehu &asrat,2015	1.305	0.546	3.123	0.599	0.549	12.42
Defersha and Belete,21	0.702	0.347	1.424	-0.980	0.327	13.52
Melkamu,2016	0.583	0.220	1.544	-1.086	0.277	11.73
Setargew et al,.2012	1.333	0.431	4.128	0.499	0.618	10.70
Tsega & derso, 2015	3.407	0.933	12.439	1.856	0.064	9.66
Wudeneh,2018	0.171	0.104	0.281	-6.983	0.000	14.79
Yihunie et al., 2019	0.629	0.330	1.197	-1.412	0.158	13.92
	0.701	0.373	1.318	-1.103	0.270	

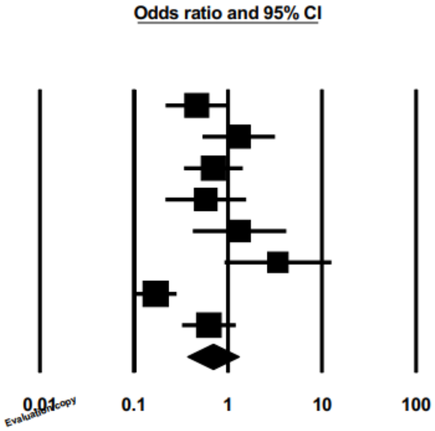

b)

Study name	Odds ratio	Lower limit	Upper limit	Z-Value	p-Value	Relative weight
Abera.M et al., 2020	0.395	0.187	0.837	-2.426	0.015	13.15
Alemayehu &asrat,2015	1.517	0.672	3.428	1.003	0.316	12.99
Defersha and Belete,21	1.640	0.751	3.584	1.241	0.215	13.07
Melkamu,2016	10.960	2.571	46.723	3.236	0.001	11.12
Setargew et al,.2012	2.830	0.710	11.284	1.474	0.140	11.34
Tsega & derso, 2015	5.022	1.310	19.250	2.354	0.019	11.46
Wudeneh,2018	0.091	0.051	0.162	-8.136	0.000	13.52
Yihunie et al., 2019	0.844	0.436	1.632	-0.505	0.614	13.35
	1.245	0.434	3.571	0.408	0.683	

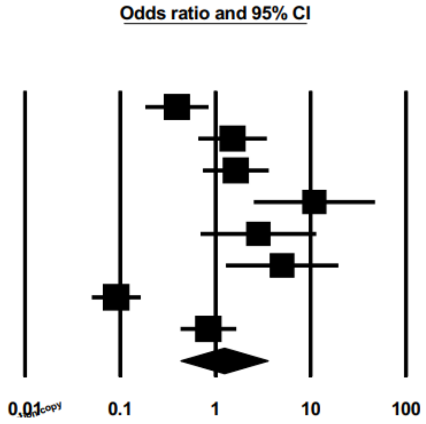

c)

Study name	Odds ratio	Lower limit	Upper limit	Z-Value	p-Value	Relative weight
Abera.M et al., 2020	0.846	0.453	1.579	-0.526	0.599	14.04
Alemayehu &asrat,2015	1.162	0.563	2.398	0.407	0.684	13.28
Defersha and Belete,21	2.335	1.246	4.375	2.647	0.008	14.01
Melkamu,2016	18.804	5.609	63.042	4.754	0.000	9.65
Setargew et al,.2012	2.122	0.503	8.956	1.024	0.306	8.19
Tsega & derso, 2015	1.474	0.636	3.418	0.904	0.366	12.37
Wudeneh,2018	0.533	0.286	0.994	-1.980	0.048	14.04
Yihunie et al., 2019	1.342	0.757	2.378	1.008	0.313	14.42
	1.587	0.893	2.822	1.573	0.116	

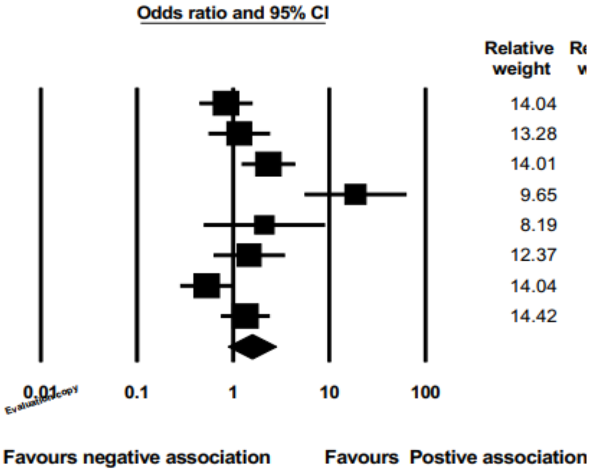

Fig.16 Odds ratio forest plots for the relationship of age categories with (2 years vs. 2-5) years (a), (<2 years vs.>5) years (b), and (2-5 vs.>5) years(c) categories with the prevalence of bovine schistosomiasis.

Publication bias of risk of age in bovine schistosomais; the absence of published studies was demonstrated by the symmetrical distribution of the funnel plot (Figure). This was confirmed by Egger's regression test (b= -1.66, P = 0.43; Fig.17), which showed no evidence of publication bias across studies to animal ages.

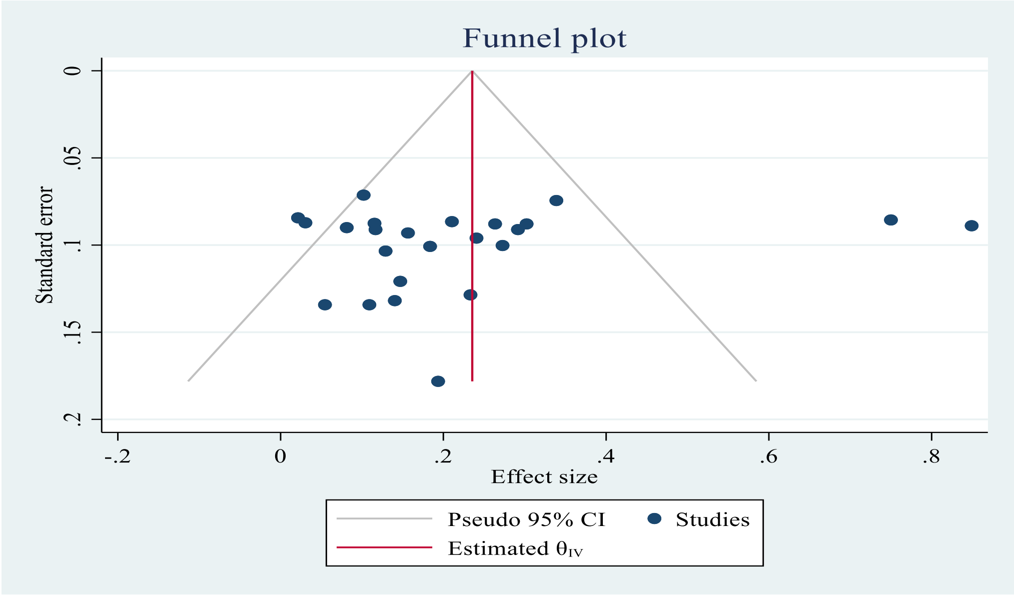

Fig.17 The funnel plot was used to assess publication bias in studies on the prevalence of bovine schistosomiasis.

Association extensive vs. semi-intensive management system with schistosomiasis

Four of the identified studies [13, 24, 25, 28] examined the association between the prevalence of bovine schistosomiasis and the management system of the animals. Two of four [13, 24] had shown a statically significant association of the management system with bovine schistosomiasis among cattle. An extensive management system had an effect on the prevalence of bovine schistosomiasis whereas the remaining two studies [25, 28] have revealed that the management system had no effect on the prevalence of bovine schistosomiasis.

Therefore, Pooled odds ratio analysis was conducted to investigate the relationship between the management system and the occurrence of schistosomiasis infection among cattle. The results in Fig. (19) Show that the result of pooled odds ratio showed no significant association (OR=0.809, 95% CI;

0.165 to 3.967; *P*=0.793) across the studies. However, there were a difference prevalence estimation in different management systems, intensive and extensive management systems.

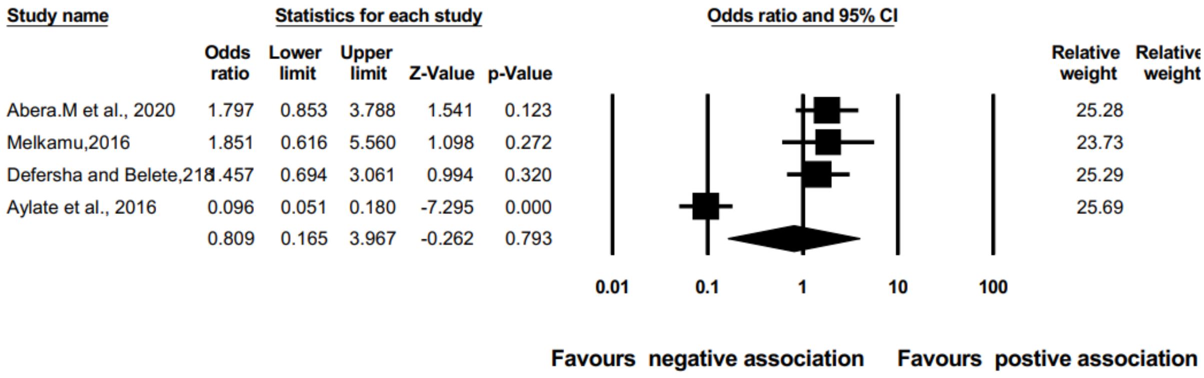

Fig.18 Pooled odds ratio for a management system with the prevalence of bovine schistosomiasis from random effects meta-analysis of four studies

The funnel plot (Fig.19) and the egger's regression asymmetry coefficient [b =0.63] (95 % CI = -3.09, 6.6; p > 0.05, = 0.84). There was no evidence of publication bias, and no conceptual missing study was reported.

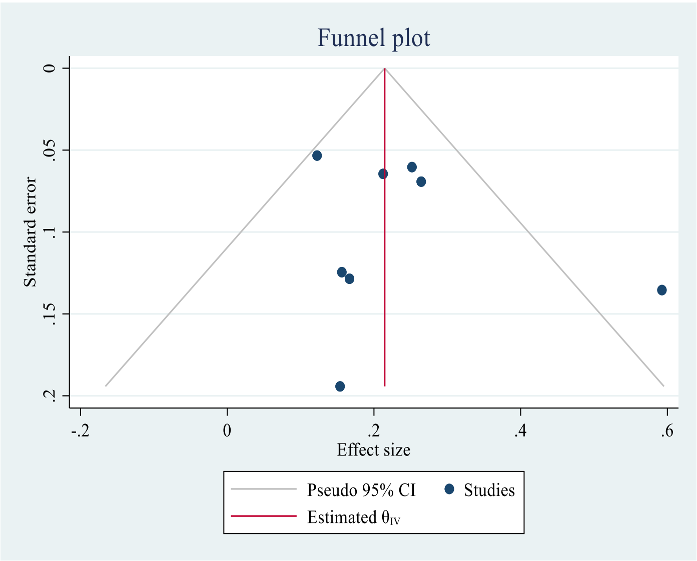

Fig. 19 Funnel plots of the standard error by mean effect size estimates of bovine schistosomiasis in the different management systems

Table 4 summarizes the heterogeneity and effect sizes between hypothesized risk factors and bovine schistosomiasis prevalence

Risk factors	Group	df	Q	P	tau^2	% I^2	H^2	ES	N
**Body condition**	Poor	9	28.95	0.00	0.03	76.87	4.32	0.33	10
	Medium	9	5.27	0.81	0.00	0.00	1.00	0.16	
	Good	9	3.16	0.95	0.00	0.00	1.00	0.09	
**Overall**		29	75.85	0.00	0.015	61.15	1.00	0.20	
**sex**	Male	17	115.76	0.00	0.030	81.28	5.34	0.20	18
	Female	17	108.85	0.00	0.026	83.44	6.04	0.21	
**overall**		35	224.64	0.00	0.027	82.16	5.61	0.20	
**Breed**	Local	8	9.85	0.276	0.001	14.25	1.17	0.18	9
	cross	8	9.84	0.277	0.004	22.27	1.29	0.17	
**Overall**		17	19.84	0.283	0.001	9.29	1.10	0.18	
**Age**	<2	7	8.06	0.00	0.005	28.77	1.40	0.18	8
	2-5	7	38.67	0.327	0.036	80.82	5.21	0.28	
	>5	7	63.0	0.00	0.064	88.71	9.86	0.22	
**Overall**		23	114.84	0.00	0.033	78.49	4.65	0.23	
**managment**	Extensive	3	3.9	0.29	0.00	25.19	1.34	0.20	
	Semi - intensive	3	7.51	0.06	0.03	59.93	2.50	0.27	4
**Pooled effect**		6	12.12	0.57	0.0	34.2	1.52	0.21	

Discussion

A review and meta-analysis were carried out in Ethiopia to estimate the pooled prevalence of bovine schistosomiasis and its association with risk factors. According to the current review, which included 20 studies, the pooled prevalence of bovine Schistosomiasis in Ethiopia was 24 percent (95% CI: 0.17 to 0.31). This pooled prevalence was lower than various individual studies conducted in Ethiopia, of which 37% [12], 26.8% [10], 60.3% [33], 60.4% [23] and 45.7% [36]. This low prevalence obtained in this analysis can also be attributed to the lower prevalence in the majority of included individual studies.

Conversely, this pooled prevalence was much higher than 11% [11], 17% [30], 14% [31], 18% [32], 5.8% [22], 18.2%, [28], 10% [20], 18% [26], 16.3% [27], 16.8% [34], and 18.5% . Various research results from various regions of the country showed that the prevalence of Schistosomiasis varies greatly between regions. It has been reported that the rate of Schistosoma infection in cattle increases during the rainy season due to an abundance of snails and favorable land conditions that allow the intermediate host to survive compared to the dry season [37]. However, the discrepancy in the prevalence of bovine schistosomiasis between this pooled one and the different individual studies conducted in different times and regions of Ethiopia was associated with environmental sanitation; the availability of water, and the health of animals, and the socioeconomic level of places are all factors to consider. Furthermore, the disparities noted above could be attributable to methodological variances (i.e., data analysis, and sampling of study participants).

In light of global concern, the pooled prevalence of bovine schistosomiasis in the current systematic review and meta-analysis finding was also higher than the sero-prevalence report of 6.5% inYola Metropolis, Adamawa State by [38], and 10.0% in Maiduguri Metropolis and Jere Local Government Areas of Borno State [39], in Nigeria. This difference in prevalence may be partly due to the difference in livestock management practices and agricultural land use plans.

The present study revealed that poor body condition had significant impact ($p<0.05$) on the prevalence of bovine Schistosomiasis. In the included studies, [34] reported a similar finding. In the present systematic and meta-analysis, cattle having poor body condition (33%), medium body condition (16%) and the prevalence of bovine schistosomiasis was found to be 9 percent in good body condition. The odds ratio that has been pooled of bovine schistosomiasis in poor body conditions showed that 4.92 times

(OR=4.92; p=0.00) than the animals' good physical condition. On the other hand, the pooled odd ratio of bovine schistosomiasis in medium body conditions showed that 1.757 times (OR=1.757; *p*=0.00) was greater than the good body condition of the animals. This study was supported by the included studies [13, 24, 36, 25, 26, 28] whereas contradicts the two included studies ([22, 32]. This outcome is also consistent with Lulie and Guadu's [39] study of the prevalence of Schistosoma.

As a result of this meta-analysis, the infection rate of Bovneschistosomiasis was higher in animals with a low body condition score have than those with a medium or good body condition score. Likewse, Fromsa, et al [40], animals with a poor body condition score had a higher infection rate of bovine schistosomiasis. This condition could be linked to poor body condition and emaciated animals becoming compromised and susceptible, which could be caused by insufficient nutrient intake, secondary complications, and/or mixed parasite infection [41].

Schistosomiasis was found in 20% of male animals and 27% of female animals, respectively. However, no statistically significant difference (P>0.05) was found between the sexes (Table 4). A similar finding was conducted by [11, 13, 24], the pooled odds ratio of male to female also confirmed that no significant difference (OR=1.007; p= 0.927) between infection incidence of bovine schistosomiasis and animal gender. This may be due to exposure of both sex categories in similar grazing areas and same watering system and finally equal chance of developing the disease [12].

There was no strong significant association (p>0.05) between the prevalence of *Bovine Schistosomiasis* and the animal management system, according to the quantitative evaluation section of this manuscript. This may be because just a few researches on the relationship between management strategies and schistosomiasis infection rates were included in the studies. Another reason could be this parasite is chronic and it produces a restricted number of eggs. Furthermore, the only method of diagnosis was a microscopic investigation of eggs. Despite no significant difference that links bovine schistosomiasis to the management system, semi-intensive management 27 % had a higher infection rate than extensive management 20%. This could be because animals that have been extensively managed animals develop a high level of immunity as a result of repeated natural exposure [42]. Animals reared in a semi-intensive management system, on the other hand, become prone to infection because they lack acquired immunity to the disease.

Limitation of the study

The study has some limitations. The first limitation could be the investigation (parasitic recovery) method was only a coprological examination. So, the quality of the studies may not be reported as real results, false negative results may be recorded. The second drawback; the greater number of the included articles was obtained from the Amhara regional state whereas the smallest number in SNNPR and no study was obtained from Benishangul Gumuz, Gambella, and Harari. Therefore, this unequal distribution of studies throughout the country may lead to a biased estimation of this finding. The third limitation studies are included in meta-analysis in case of associated risk factors and subgroup analysis was not sufficient in number. The other limitation, even after subgroup analysis, the original data revealed a considerable degree of heterogeneity between trials. As a result, this finding may not necessarily reflect the current condition in Ethiopia's entire region.

Conclusions

It is the first meta-analysis and systematic review attempt to assess the pooled prevalence of bovine schistosomiasis in Ethiopia that we are aware of. The basic guideline for Preferred Reporting Items for Systematic Reviews and Meta-analysis was followed in this systematic review and meta-analysis (PRISMA). The prevalence of bovine schistosomiasis was higher in this systematic review and meta-analysis than in individual investigations. Hence, this figure revealed that schistosomiasis is a widely distributed and economically important disease of livestock in Ethiopia. The prevalence discrepancy of reports may be explained by the difference in location, agroecology, method and season of study. The heterogeneity between various prevalence studies was large which may be due to the number of sample sizes, study regions and study year. Even, in subgroup analysis high degree of variability was observed between regions, study year as well as sample size among included studies. In the risk factor studies, the occurrence of bovine schistosmiasis was strongly associated with body condition of the animals. Based on some empirical findings and the current pooled prevalence estimate, Ethiopian public health institutions and other responsible bodies should focus on the substantial reduction of this high level of prevalence because it has public health impact.

Funding

Not applicable

Competing interests

With this review, we have no competing interests.

Availablity of data and materials

This article contains most of the data that was analyzed during the research and some of the materals are included in the supporting informations

Supporting information

S1 File. Meta- regression (DOC)
https://docs.google.com/document/d/1yDmO41lDP9xI2trsi3MzKmte3-XPkfvRUyYX7rWBiZc/edit?usp=sharing

S2 figs. 1, 2 and 3 Bubble plot.
https://docs.google.com/document/d/1XVN8h4qgnvZxhh4yBpynAR1MwCoGfET9y6wirIyJabc/edit?usp=sharing

S4 file. The PRISMA- Checklist (pdf).
https://docs.google.com/document/d/1A3IO5EguivxXPZAgXgKmO9xzuvaBl9wQ8rXV37dDC-8/edit?usp=sharing

S5 dataset.
https://docs.google.com/spreadsheets/d/1umTniOoMi-3DA7eDqGfmmy6vt0j-UOlU3lmCKDmI-e4/edit?usp=sharing

Acknowledgment

We thank University of Gondar, for providing some of the materials and important pieces of trainings.

Authors' contributions

Conceptualization: Melkie Dagnaw, Tsegaw fentie, Bihonegn wodajnew, and Nigist Bizu.

Software & **meta-analysis:** Melkie Dagnaw and Tsegaw Fentie.

Methodology: Atsede Solomon, Firdyawukal Abuhy and Melkie Dagnaw.

Writing reveiew and edting: Ahmed Abi, Melkie Dagnaw, and Nigist Bizu, Melkie Dagnaw, Atsede Solomon, Bihonegn wodajnew and Firdyawukal Abuhy

Validation: All authors participated in all Procedures of preparation, writing and approved the final manuscript.

References

1. Parja, C.S. Textbook of Parasitology, protozoology and helminthology. 2nd ed. Channai; 2004; 141-256.
2. Lefevre, C., Blancou, J., Chermette, R. & Uilenberg, G. Infectious disease of livestock. *Paris. Lavoizer;* 2010*;* 2(5)**,** 1699-1703.
3. Hailu, B., Shibru, T., Leykun, J. Schistosomiasis in Ethiopia and Eritrea. 3rded. Institute of pathobiology, Addis Ababa University: 1998; 123-189.
4. Singh A, Singh A, Chaudhri SS. Visceral schistosomiasis of domestic animals in India: humoral immune status of infected cattle, sheep and goats against major polypeptide antigens of Schistosoma indicum and S. spindale. Parasite Immunology. 2004 Apr; 26(4):167-75

.5. Hambali IU, Adamu NB, Ahmed MI, Bokko P, Mbaya AW, Tijjani AO, Biu AA, Jesse FF, Ambali AG. Sero-prevalence of Schistosoma species in cattle in Maiduguri metropolis and Jere local government areas of Borno State, Nigeria. Journal of Advanced Veterinary and Animal Research. 2016 Apr 15; 3(1):56-61.

6. Oliveira G, Rodrigues NB, Romanha AJ, Bahia D. Genome and genomics of schistosomes. Canadian Journal of Zoology. 2004 Feb 1;82(2):375-90.
7. Trottein F, Godin C, Pierce RJ, Sellin B, Taylor MG, Gorillot I, Silva MS, Lecocq JP, Capron A. Inter-species variation of schistosome 28-kDa glutathione S-transferases. Molecular and biochemical parasitology. 1992 Aug 1; 54(1):63-72.
8. Li S, Qian Y, Yang K *et al*. (2012): Successful outcome of an integrated strategy for the reduction of schistosomiasis transmission in an endemically complex area. *Geosp Hlth;* 2012; 6 (2): 215-220
9. Urquhart GM, Armour J, Duncan JL, Jennings FW. Veterinary helminthology: veterinary parasitology. New York: Churchill: Livingstone Inc. 1996.
10. Kerie Y, Seyoum Z. Bovine and ovine schistosomiasis: prevalence and associated host factors in selected sites of South Achefer district, northwest Ethiopia. The Thai Journal of Veterinary Medicine. 2016 Dec 1;46(4):561.
11. Alemayehu A, Asrat M. Crossectional Study on Prevalence of Bovine Schistosomiosis and its Associated Risk Factors in Dangila District, Amhara National Regional State, Ethiopia. Journal of Animal Research. 2015 Sep 1;5(3):397.
12. Habtamu A, Mariam SW. Repeated simple sedimentation technique and prevalence of bovine schistosomosis in selected sites of Bahir Dar woreda. Ethiopian Veterinary Journal. 2011; 15(1).

13. Aylate A, Hussen M, Tilahun A, Kiros A. A cross-sectional study on bovine schistosomiasis in and around Kemissie, Dawa Cheffa District. Journal of Veterinary Medicine and Animal Health. 2017 Apr 3; 9(4):72-7.

14. Moher D, Liberati A, Tetzlaff J, Altman DG, Prisma Group. Preferred reporting items for systematic reviews and meta-analyses: the PRISMA statement. International journal of surgery (London, England). 2010;8(5):336-41..

15. Page MJ, McKenzie JE, Bossuyt PM, Boutron I, Hoffmann TC, Mulrow CD, et al. The PRISMA 2020 statement: an updated guideline for reporting systematic reviews. BMJ 2021; 372:n71. doi: 10.1136/bmj.n71

16. Higgins JP, Thompson SG. Quantifying heterogeneity in a meta-analysis. Statistics in medicine. 2002 Jun 15; 21(11):1539-58.

17. Jin ZC, Zhou XH, He J. Statistical methods for dealing with publication bias in meta-analysis. Statistics in Medicine. 2015 Jan 30;34(2):343-60..

18. Borenstein M, Cooper H, Hedges L, Valentine J. Effect sizes for continuous data. The handbook of research synthesis and meta-analysis. 2009 Feb 5; 2:221-35.

19. Shi X, Nie C, Shi S, Wang T, Yang H, Zhou Y, Song X. Effect comparison between Egger's test and Begg's test in publication bias diagnosis in meta-analyses: evidence from a pilot survey. Int J Res Stud Biosci. 2017; 5(5):14-20.

20. Setargew, M., Fentahun, T. & Chanie, M. Prevalence of Bovine ShistosomiasisinFogera District, South Gondar Zone, Amhara National Regional State, Northwest Ethiopia; 2012.

21. Hansen J, Perry B. Epidemiology, Diagnosis and Control of Helminth parasite of Ruminants. Hand book of animal production and health division, FAO, Rome, Italy, p.171.Int. Hlth; 1994; 3: 372

22. Kebede, A., Dugassa, J., Haile, G. & Wakjira, B. (2018). Prevalence of bovine of schistosomosis in and around Nekemte, East Wollega zone, Western Ethiopia. Journal of Veterinary Medicine and Animal Health. 2018 May 31;10(5):123-723.

23. Yeneneh A, Kebede H, Fentahun T, Chanie M. Prevalence of cattle flukes infection at Andassa Livestock Research Center in the northwest of Ethiopia. In Veterinary Research Forum 2012 (Vol. 3, No. 2, p. 85).

24. Abera.M. Fentahun.G. Peer.F. Prevalence of bovine schistosomiasis and its associated risk factors in and around Bahir Dar Town, Northwest Ethiopia. Daagu International Journal of Basic & Applied Research DIJBAR. 2020; 2(1): (66-74).

25. Defersha T, Belete B. The Neglected Infectioufs Disease, Bovine Schistosomiasis: Prevalence and Associated Risk Factors for its Occurrence among Cattle in the North Gulf of Lake Tana, Northwest Ethiopia. J Vet Med Health. 2018;2:112.
26. Tsega M, Derso S. Prevalence of bovine schistosomiasis and its associated risk factor in and around Debre Tabor town, northwest of Ethiopia. Europ J Biol Sci. 2015;7:108-3.
27. Wudeneh A. A Study on Prevalence of Bovine Schistosomiasis in Fogera Woreda, North Western of Ethiopia. pathology. 2018;14:16
28. Melkamu S. Study on the prevalence and associated risk factors of bovine and human schistosomiasis in Bahir Dar and its surrounding areas. Journal of Animal Research. 2016 Dec 1;6(6):967..
29. Yihunie A, Urga B, Alebie G. Prevalence and risk factors of bovine schistosomiasis in Northwestern Ethiopia. BMC veterinary research. 2019 Dec;15(1):1-5.
30. Aragaw K, Tilahun H. Coprological study of trematode infections and associated host risk factors in cattle during the dry season in and around Bahir Dar, Northwest Ethiopia. Veterinary and Animal Science. 2019 Jun 1;7:100041.
31. Chanie M, Dejen B, Fentahun T. Prevalence of cattle schistosomiasis and associated risk factors in Fogera cattle, South Gondar zone, Amhara National Regional State, Ethiopia. Journal of Advanced Veterinary Research. 2012 Jul 1;2(3):153-6.
32. Gebremeskel.K A, Tayelgn.S. and Mekuria.A. Prevalence and associated risk factors of bovine schistosomiasis in Northwestern Ethiopia.Wrld. Vet. J. 2017;7(1):01-4: *http://dx.doi.org/10.5455/wvj.20170487*
33. Merawe M, Kassaw Amssalu YH, Afera B. Intestinal Schistosomiasis of Bovine and Ovine in Fogera District, South Gonder Zone, Amhara National Regional State, Ethiopia. Young. 2014;94(9):9-57.
34. Mohammed K, Suwaiba M, Spencer T, Nataala S, Ashcroft O, Nuhu A, Asiya UI. Prevalence of urinary schistosomiasis among primary school children in Kwalkwalawa area, Sokoto state, north-western Nigeria. Asian J Res Med Pharm Sci. 2018;3(1):1-0.
35. Shiferaw MB, Deressa FB. Prevalence and Associated Risk Factors of Bovine Schistosomiasis in and Around Bakko Town, west Shoa Zone, Oromia, Ethiopia. Global J Sci Front Res. 2017;500.
36. Bertualem.T, Kassa.T and Birhanu.T. (2017).Prevalence of Bovine flukes in Irrigation Canals of Amibara District, Afar Region, North-East Ethiopia *Researcher* ;9(4):89-94]. ISSN 1553-9865 (print); ISSN 2163-8950.http://www.sciencepub.net/researche

37. Kahn. CM. The Merck Veterinary Manual.10th Edition. White House Station, New Jersey. Merck & Co, Inc; 2011; 273-1036.

38. Zamdayu NM, Qadeer MA, Francis MI, Tillo IM. Seroprevalence of Bovine Schistosomosis in Yola Metropolis, Adamawa State, Nigeria. Alexandria Journal for Veterinary Sciences. 2019 Apr 1;60(2).

39. Lulie B, Guadu T. Bovine schistosomiasis: A threat in public health perspective in Bahir Dar town, northwest Ethiopia. Acta Parasitologica Globalis. 2014;5(1):1-6.

40. Fromsa A, Meharenet B, Mekibib B. Major trematode infections of cattle slaughtered at Jimma Municipality Abattoir and the occurrence of the intermediate hosts in selected water bodies of the zone. Journal of Animal and Veterinary Advances. 2011;10(12):1592-7.

41. Niaz S, Tanveer A, Qureshi AW. Prevalence of schistosomiasis in cows and buffaloes at different sites of Punjab Pakistan and its relation to temperature, relative humidity, rainfall and pan evaporation. Pakistan Journal of Science. 2010;62(4):242-9.

42. Cheng. TC. General Parasitology. 2nd edition. Academic Press, Medical University of South Carolina, Charlton; 1986; 332-342

MJ, McKenzie JE, Bossuyt PM, Boutron I, Hoffmann TC, Mulrow CD, et al. The PRISMA 2020 statement: an updated guideline for reporting systematic reviews. BMJ 2021; 372:n71. Doi: 10.1136/bmj.n71 visit:http://www.prisma-statement.org/

Click here to access/download
Supporting Information
PRISMA_2020_checklist-ref.docx
