# Supplementary material for: Epidemiology of bovine schistosomiasis and associated risk factors in Ethiopia: A systematic review with meta-analysis of published articles, 2008–2018
Source: PLoS One. 2023 Jul 31;18(7):e0283691. doi: 10.1371/journal.pone.0283691 (PMC10389744; doi:10.1371/journal.pone.0283691)
Supplement: Attachment — Submitted filename: Reviewers comments.docx [file pone.0283691.s008.docx]

1. This is critical work in terms of providing evidence-based information for bovine schistosomiasis, particularly in this era of *Schistosoma* hybridization. As a result, authors should clearly state the purpose of this review. The absence of a systematic review and meta-analysis of bovine schistosomiasis in Ethiopia is not a compelling reason to conduct this review. The reason for this could be to provide information that can be used in the planning and design of *Schistosoma* interventions in Ethiopia, as the world aims to eliminate schistosomiasis as a public health problem by 2030.
2. The contribution of each author should be provided in each section of this review, particularly during article searching, screening, data extraction, and article quality assessment. This will demonstrate the caliber of their work.
3. There are so many contradictory statements in the review. Authors should paraphrase them to provide clear meanings.
4. There is some statement repetition. Authors should make sure that each statement appears only in its appropriate section.
5. There are so many unnecessary statements in the methodology section. These should be omitted or placed in the introduction or discussion sections.
6. Authors should conduct quality assessments using any of the available standard guidelines and appropriately present the results.
7. The results of the meta-analysis and the bias assessment should be separated by the authors. To ensure a smooth flow of information, the results of meta-analysis can be combined with those of subgroup analysis.
8. To confirm the funnel plot result, I recommend authors present only one test result, choosing between Egger's and Begg's tests. Also, publication bias and quality assessment of all included studies should be done once. No need of the repeated assessments. Studies presented low quality should be omitted from analysis.
9. The data analysis section should clearly state the subgroups (factors) that were involved during subgroup analysis and categorization of each subgroup (factor). Furthermore, the categories must be distinct.
10. Subgroup analysis results were limited and poorly presented. The authors did not present the pooled prevalence of bovine schistosomiasis in each category of subgroup with a significant contribution of heterogeneity, nor did they present the amount of heterogeneity contributed by each factor (R^2^).
11. There are far too many repetitions of the results in the discussion section, rather than discussions of the results.
12. The conclusion section should be revised by providing statements that generalize all key findings. There is no need to discuss them.
13. A native English speaker or a professional English language editor should revise the entire manuscript.
14. Find other comments in the pdf file.
